# Correction for the pupil size artifact improves the measurement of fixation drift with a head-mounted pupil tracker

**DOI:** 10.3758/s13428-026-03103-z

**Published:** 2026-07-14

**Authors:** Thomas Eggert

**Affiliations:** 1 Department of Neurology, LMU University Hospital, LMU Munich, Munich, Germany; 2 Planegg/Martinsried, Germany

**Keywords:** Human, Oculomotor, Fixation drift, Video-oculography, Pupil artifact

## Abstract

The exactness of pupil-based video-oculography (pVOG) is limited by the pupil size artifact (PSA), involving shifts in pupil position with changes in pupil diameter. This study investigates PSA’s effects on fixation drift measurement and compares two correction methods. The first, the “interpolation method,” creates calibrations for different pupil sizes by adjusting illumination and interpolating between them based on the current pupil diameter. The second, the “regression method,” uses natural fluctuations in pupil size during fixation to estimate PSA gain via regression, then corrects pupil position accordingly. Here, the regression method was optimized for correcting fixation drift measurements by removing microsaccades and frequency components below 1 Hz and above 15 Hz. PSA was compared with size-related pupil displacements relative to the limbus to better investigate its cause. Pupil dilation caused downward and temporal shifts (PSA gains: 0.23 deg/mm horizontally, 0.62 deg/mm vertically). The PSA affected vertical fixation drifts (*R*^2^ = 14%) more strongly than horizontal drifts (*R*^2^ = 1.5%). The mean and precision of PSA gain estimates did not differ between methods. Both reduced the determination of fixation drift by pupil diameter changes (*R*^2^). PSA stability over 12 min was confirmed in 77% of cases. Power spectral density analysis supported the effectiveness of the regression correction, aligning with previous findings. PSA is probably caused mainly by the shift of the pupil relative to the limbus, as it did not differ significantly from the shift in camera coordinates. Overall, the regression method is a promising technique for correcting PSA in pVOG to improve fixation drift measurements.

**Supplementary Information:**

The online version contains supplementary material available at 10.3758/s13428-026-03103-z.

## Introduction

During fixation, and between saccades and microsaccades, the eyes show microscopic movements called fixation drifts. Even without systematic drifts due to pathological deficits in gaze holding or vestibular imbalances, alternating components of these microscopic drifts occur during steady fixation with frequency components up to 90 Hz (Carpenter, 1988; Ditchburn & Ginsborg, 1953; Findlay, 1971; Ko et al., 2016). In the current study, which focuses on the measurement of these alternating components within a frequency range between 1 and 15 Hz, these components are called “oscillatory fixation drift” or just “fixation drift” for brevity. Similar to the tremor of the arm or hand, fixation drift also represents a type of physiological tremor (Hallett, 1998; Marshall & Walsh, 1956). Exaggerated physiological tremors occur in anxiety, stress, fatigue, hypothermia, or in the presence of drugs (Hallett, 1998).

Fixation drift has predominantly been addressed using the scleral search coil (Collewijn et al., 1975) and the dual Purkinje image (Crane & Steele, 1985) (DPI), which provide more precise measurements than pVOG. Unfortunately, the scleral search coil is semi-invasive and requires local anesthesia of the cornea. The DPI technique (Crane & Steele, 1985) is difficult to handle and maintain because of its complex servo feedback, used to keep the optical detectors centered on the optical axis of the eye. For these reasons, pVOG has become the predominant method for quantifying eye movements, particularly for binocular measurements.

A major reason why pVOG has thus far been considered unsuitable for investigating fixation drift in healthy subjects is the pupil-size artifact (PSA). During fixation on a stationary target, the PSA manifests in shifts of the pupil position in the camera image caused by changes in pupil size rather than eye rotation (Choe et al., 2016; Drewes et al., 2014; Hooge et al., 2024; Hooge et al., 2019; Hooge et al., 2021; Jaschinski, 2016; Wildenmann & Schaeffel, 2013; Wyatt, 2010). This artifact is thought to reflect small anisotropies in pupil contraction and dilation, leading to a displacement of the pupil relative to the limbus (Wyatt, 1995). However, a direct quantitative comparison between the PSA and the displacements of the pupil relative to the limbus has not been undertaken.

Pupil size not only changes with luminance but also shows spontaneous activity, which is thought to be related to mental activity or mental state (Ariel & Castel, 2014; De Gee et al., 2014; Kahneman & Beatty, 1966; Naber & Nakayama, 2013; Nassar et al., 2012). Spontaneous changes of the pupil diameter have a power spectral density with a 20-dB cutoff frequency of about 2.5 Hz (Peysakhovich et al., 2015). The PSA is therefore expected to contaminate measurements of fixation drifts in that range of frequencies. Consequently, accounting for the PSA is crucial when measuring fixation drift accurately with pVOG. Two different methods have been proposed previously to correct pVOG measurements for the PSA (*PSA correction*).

The first method, referred to as the *interpolation method* here, was proposed by Drewes et al. (2014). It manipulates the pupil diameter during fixation calibration, allowing the eye position to be approximated as a function of not only the horizontal and vertical pupil positions in the camera image, but also the pupil diameter. In a recording following the calibration measurement, this calibration is then applied using the currently measured pupil diameter, thus correcting for the PSA. This method is very general, as it can also correct for dependencies of the PSA on the viewing direction, depending on the complexity of the selected calibration function. The disadvantage of this approach is that it requires an extended calibration session.

The second method, called the *regression method* here, was inspired by Choe et al. (2016). It uses spontaneous changes of pupil diameter during fixation to estimate the PSA via a regression analysis between calibrated eye position and pupil diameter. In contrast to the interpolation method, the regression method does not require a separate calibration measurement. The PSA is estimated and corrected within the same data set. Hence, the regression method can adapt to possible changes in PSA between data sets. The regression method estimates the PSA only over a small range of eye positions and ignores potential position dependencies in the PSA. However, this simplification does not cause critical errors as long as fixation drift is only examined around a single eye position (e.g., straight ahead). The precision of this method is limited by the actual eye drift during fixation, as these movements act as noise in the regression analysis of eye position on pupil diameter.

Whereas previous studies focused on the effects of the PSA on the standard deviation of the fixation error between successive refixations of the same target (Choe et al., 2016; Hooge et al., 2024), the current study is mainly interested in the effect of the PSA on the estimation of fixation drift obtained from within short fixation intervals. The main difference between these two measures is that successive refixations are contaminated by the low-frequency components of the PSA (< 1 Hz), whereas oscillatory fixation drifts are subject to PSA components with higher frequency. Therefore, the current study optimizes the regression method of Choe et al. (2016) for PSA correction for fixation drift measurements in the following way. Microsaccades and saccades were eliminated before the regression. To further improve the precision of the evaluation of the PSA in the frequency range of interest, eye positions were filtered to exclude frequencies below 1 Hz. Frequencies above 15 Hz were removed to reduce the influence of the noise characteristic of pVOG. In this way, the estimation and compensation of the PSA were optimized for the range at which fixation drift is most accurately measured (see Discussion). Correction was achieved by subtracting the estimated PSA from the calibrated eye position.

### Objectives

The present study has two main objectives. First, it aims to compare the two methods for estimating and correcting artificial fixation drifts induced by the PSA. To this end, Experiment 1, consisting of a calibration paradigm (a visually guided saccade task with targets in various positions) and a fixation condition on a centered white cross, was performed. The *PSA estimate* is defined as artifact-induced gaze shift (deg) corrected by the respective method. It should be noted that both models considered in the current study assume a static mapping between pupil diameter and the PSA, and that the dynamics of artificial fixation drifts explained by these static models are completely determined by the dynamics of the pupil diameter. The most fundamental static parameter of these models is the *PSA gain*, expressed in units of deg/mm. It is defined as the slope of the linear approximation of the static function that models the PSA depending on pupil diameter. For known pupil diameter and linear PSA models, the PSA estimate is already determined by the estimate of the PSA gain. Therefore, and because the current study uses only linear PSA models, the terms “PSA gain estimate” and “PSA estimate” are treated as largely synonymous here. The PSA gain was estimated from the data using three different approaches:

(I) by the interpolation method, analyzing the calibration recording
(II) by the regression method, analyzing the calibration recording.
(III) by the regression method, analyzing the fixation recording.

Systematic differences between the two methods were evaluated by comparing the PSA gains (I) and (II), i.e., with data from the same recording. To assess the precision of the PSA gain estimate, the within-subject standard deviation of the estimated PSA gain was evaluated and compared between all three approaches. The temporal stability of the PSA was quantified by comparing the PSA gains (II) and (III), i.e., with the same method but with two subsequent recordings. To evaluate the performance of each method, the proportion of variance in apparent fixation drift (before correction) and fixation drift (after correction) that was explained by changes in pupil size (*R*^2^) was calculated. The difference between these two *R*^2^ values was used as a measure of the efficiency of the correction method.

The second aim of the present work is to compare the PSA with the movements of the pupil relative to the limbus. This comparison is important because both proposed methods aim to eliminate the effect of pupil size on measured eye position. They are therefore based on the assumption that changes in pupil size do not actually have any influence on the eye position during fixation of a head-fixed target. If this assumption is correct, the PSA should be identical to the movement of the pupil relative to the limbus (Wyatt, 1995), since the limbus is firmly attached to the eyeball. This is also supported by the finding of Strauch and Naber (2022) that the outer areas of the iris undergo greater elastic deformation during pupil dilation and constriction than the areas near the limbus. Because the pVOG used in this study could not evaluate the center of the limbus, the same calibration paradigm as in Experiment 1 was performed again in Experiment 2, with the video stream used by the pVOG system saved to disk for offline analysis. Only the videos of the left eye were recorded and analyzed. Based on this video, the pupil was localized relative to the limbus using a specially developed software under comprehensive manual control.

## Materials and methods

### Participants

Fourteen test subjects (age: 36.2 ± 10.7, nine females, five males) participated in Experiment 1. Of these, 11 (age 38.2 ± 11.7) also completed Experiment 2. All subjects had normal or corrected-to-normal vision (Snellen acuity better than “6/6”). The recording quality of the applied pVOG depended largely on the pupil margin not covered by the eyelid. Therefore, only subjects who opened their eyes wide enough and had good control over their blink reflex were recruited. All participants were recruited from the employees of the University Hospital of the Ludwig-Maximilians University Munich and gave their written informed consent. The experimental procedure was in accordance with the Declaration of Helsinki and was approved by the Ethics Committee of the Medical Faculty of the Ludwig-Maximilians University Munich (280–10).

### Apparatus

Eye movements were recorded with a binocular video-based head-mounted pupil tracker (Schneider et al., 2009) running at a frequency of 220 Hz. Each eye was recorded by its own camera. The optical path of the camera was directed at the eye via a semi-transparent mirror at an angle of about 30 degrees from below. This system (EyeSeeCam®, EyeSeeTec GmbH, Munich, Germany) evaluated, for each eye and for each sample, the pupil position in the image coordinates of the cameras at a resolution of about 0.01 deg (specification of the manufacturer, defined as RMS eye position during steady fixation after subtraction of a linear fit). In the fixation intervals of the current study, the median [IQR] of the RMS sample-to-sample difference of the unfiltered eye position data was 0.0254 [0.0130] deg in the horizontal and 0.0250 [0.0310] deg in the vertical direction. For each image frame, the EyeSeeCam® also provided the ellipse parameters of the pupil image (orientation and length of the two main axes). Only the ellipse parameters of the left eye were recorded. The system marked full blinks by missing values in the eye position traces.

Visual stimulation was presented on a video monitor (ASUS 278H; size: 59.8 × 33.6 cm; resolution: 1920 × 1080; frame rate: 100 Hz; viewing distance: 128 cm). Graphics were generated by a GeForce GTX 960 (EVGA, Brea, CA, USA) graphics card installed in a separate Linux PC (openSUSE 13.1). A custom-written C program using the OpenGL libraries controlled the graphics card.

To enable monocular viewing conditions during calibration, the graphics computer also controlled a pair of shutter glasses (PLATO, Translucent Technologies, Ontario, Canada). These were attached to the eye-tracking device’s mounting frame and positioned about 5 cm in front of the eyes (out of the cameras’ optical path). The size of the field of view through the shutter glasses was 55 × 55 deg. The graphics computer controlled the shutters via the parallel port (IEEE 1284).

The raw signals of eye position, pupil parameters, visual stimulus, and shutter glasses were synchronized by online transmission via a private network to a central recording computer running under a real-time operating system. (QNX, Ottawa, Ontario, CA). The two different sampling rates of the pupil tracker and the stimulus generator were mapped to a common data rate of 1 kHz using linear interpolation.

The monitor was the only light source in the experimental room. The head position was stabilized by a chin rest adjusted individually to align the midpoint between the two eyes with the center of the screen. Participants were instructed to keep their heads as still as possible throughout the session and not to touch the head mount of the pupil tracker. This instruction was also explicitly given for the short breaks (duration: 15 s) between the calibration paradigm and the subsequent fixation block.

### Calibration paradigm

The eye position signals were calibrated based on the pupil positions in the camera image, expressed in pixels, as provided by the EyeSeeCam® without using the pupil tracker’s built-in calibration software. The fixation targets were presented to each eye under monocular viewing conditions. To estimate the calibration’s dependence on pupil diameter, two calibration functions were created for each eye: one for a darker background (10 cd/m^2^) and one for a lighter background (200 cd/m^2^). In contrast to the study of Drewes et al. (2014), we did not establish two-dimensional calibration functions on a large (20 × 20 deg) quadratic grid with a 2D-polynom but only linear calibration functions for a small range of eccentricities between ± 7 deg on the horizontal meridian (0, ± 2.24, ± 4.47, ± 6.68 deg), and presented at an inter-target interval of 3 s (see Data analysis for the generation of the vertical calibration). Each target was presented to only one eye for a fixation interval of 1.5 s. All four monocular viewing conditions (left/right eye viewing, small/large pupil) were presented intermixed in the same calibration block. The entire calibration paradigm contained 192 = 4 × 48 fixation periods and lasted approximately 5 min. Figure 1 shows this horizontal calibration paradigm.

**Fig. 1:**
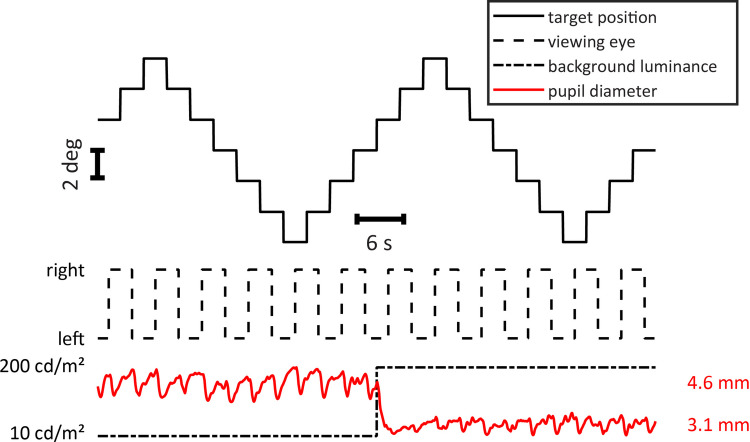
Calibration paradigm under alternating monocular viewing conditions. Fixation targets stepped through a sequence of seven horizontal target eccentricities (*solid line*). At each target position, the viewing eye was changed (*dashed-solid line*). Background luminance changed between 10 and 200 cd/m^2^ (*dash-dotted black line*) to establish separate calibration functions for small and large pupil diameters. The *red trace line* shows the pupil diameter of one participant. In this individual, the mean diameter for bright and dark backgrounds was 3.1 and 4.6 mm, respectively. The entire calibration paradigm consisted of four repetitions of the depicted stimulus sequence

### Fixation paradigm

Eye drifts were measured during fixation on a white cross (0.09 × 0.09 deg, bar width/length = 0.1), presented at the center of the screen for a randomized interval of 1.6 ± 0.3 s. Because permanent fixation on a stationary target is very difficult to maintain, we introduced a visual discrimination task to be performed at the location of the fixation cross immediately after the fixation interval. The participants decided in an adaptive two-alternative forced choice whether a briefly (200 ms) presented Landolt-C was open to the left or to the right. The answers were given by pressing a button. Each participant performed 120 of these fixation trials in about 7 min. This decision task motivated the participants to perform the fixation accurately and to suppress blinking during the fixation interval.

### Data analysis

All data analyses were performed with custom-written software under MATLAB (R2023b).

#### Pupil diameter

The pupil diameter was evaluated as the length of the major axis of the pupil image. This measure is relatively insensitive to the perspective reduction of the area of the pupil image with eccentric eye position. Since the main axis was based on the parameters of the pupil ellipse as provided by the EyeSeeCam®, pupil diameter was first expressed in units of pixels. For conversion to units of mm, the camera magnification (*m*) was defined as the ratio between pupil position [px] and the pupil position expressed in the spatial coordinates of the object principal plane (at the location of the entrance pupil) [mm]. This ratio was computed from the optical parameters of the EyeSeeCam® (object distance: *od* = 75 mm; focal length: *fl* = 12 mm; pixel size: *ps* = 0.024 mm) according to geometrical optics:

1 $$m=\frac{fl}{ps \left(od-fl\right)}=7.9 [px/mm].$$

This value was verified by recording an artificial pupil (laser print on paper, diameter 4 mm) at an object distance of 75 mm with the EyeSeeCam®. The diameter of the image of this artificial pupil was about 32 (≈ 4 × 7.9) pixels.

#### Horizontal calibration

For each of the 48 fixations per eye (*left*/*right*) and luminance condition (*i* = 1: 200 cd/m^2^; *i* = 2: 10 cd/m^2^) of the calibration paradigm, the horizontal pupil positions (${\underline{p}}_{H, left}^{i}, { \underline{p}}_{H, right}^{i}$ in units of pixels) in the camera images were calculated as the median pupil position during the last 500 ms of the 1.5-s fixation period. The relationships between the horizontal components of the pupil position (${p}_{H, left}^{i}, {p}_{H, right}^{i}$) and eye position in head-fixed coordinates (${e}_{H, left}^{i}, { e}_{H, right}^{i}$ in units of degrees) were approximated by linear functions separately for the left and right eye and two levels of background luminance:

2 $${\widehat{p}}_{ \mathrm{H}, \dots }^{i}={o}_{ \mathrm{H}, \dots }^{i}{ + e}_{ \mathrm{H}, \dots }^{i}\bullet {s}_{ \mathrm{H},\dots }^{i} ;i\in \left\{1, 2\right\}$$

The horizontal offsets and slopes (${o}_{ H, left}^{i}, {o}_{ H, right}^{i}, {s}_{ H, left}^{i}, {s}_{ H, right}^{i}$) were determined in a linear regression minimizing the squared distance between expected and measured pupil positions:

3 $${o}_{ \mathrm{H}, \dots }^{i}, {s}_{ \mathrm{H}, \dots }^{i}=\underset{{o}_{\text{ H}, \dots }^{i}, {s}_{\text{ H}, \dots }^{i}}{\text{arg min}}\left[\sum_{n=1}^{N}{\left({p}_{ \mathrm{H}, \dots }^{i}\left(n\right)- {o}_{ \mathrm{H}, \dots }^{i}-{t}_{ \mathrm{H}, \dots }^{i}\left(n\right)\bullet {s}_{ \mathrm{H}, \dots }^{i}\right)}^{2}\right]$$

where ${t}_{H, \dots }^{i}\left(n\right)$ denotes the horizontal angular position of the *n*^th^ fixation target ($1\le n\le$ *N* = 48). Conversion of any measured pupil position ${p}_{H, \dots }(\mathrm{t})$ into eye position was achieved by using the corresponding inverse regression line (Eq. 2) as the calibration function:

4 $${e}_{\mathrm{H}, \dots }^{i}\left(t\right)={c}_{ H, \dots }^{i}+{d}_{ H, \dots }^{i}{p}_{H, \dots }(\mathrm{t}) ;i\in \left\{1, 2\right\}$$

where ${c}_{ H, \dots }^{i}$ denotes the calibration offsets and ${d}_{ H, \dots }^{i}$ the calibration gains, defined by

5 $${c}_{ H, \dots }^{i}=-\frac{{o}_{ \mathrm{H}, \dots }^{i}}{{s}_{ \mathrm{H}, \dots }^{i}}; {d}_{ H, \dots }^{i}=\frac{1}{{s}_{ \mathrm{H}, \dots }^{i}} ;i\in \left\{1, 2\right\}$$

*Vertical calibration.* The parameters of the vertical calibration functions can be determined by performing a corresponding vertical calibration procedure. However, because such a calibration measurement is time-consuming, we determined the vertical offsets and slopes (${o}_{V, left}^{i}, {o}_{V, right}^{i}, {s}_{ V, left}^{i}, {s}_{ V, right}^{i}$) from the data of the horizontal calibration paradigm as described in more detail in the Supplementary material. Briefly, the vertical offsets were computed as the average across the vertical components of the pupil positions

6 $${o}_{V, \dots }^{i}=\frac{1}{N} \sum_{n=1}^{N}{p}_{ \mathrm{V}, \dots }^{i}\left(n\right)$$

This ${o}_{V, \dots }^{i}$ is the solution of the vertical minimization problem in analogy to Eq. (3) since all vertical target positions (${t}_{ \mathrm{V}, \dots }^{i}\left(n\right)$) were zero in the horizontal calibration paradigm. The vertical calibration slopes (${s}_{ \mathrm{V}, \dots }^{i}$) were derived from the horizontal slopes (${s}_{ \mathrm{H}, \dots }^{i}$) by

7 $$\begin{array}{c}{s}_{\mathrm{V},\dots }^{1}={s}_{\mathrm{V}, \dots }^{2}=0.766 {s}_{H, \dots }^{*} \text{ with } {s}_{H, \dots }^{*}=\frac{1}{{\overline{d}}_{H, \dots }}\\ \text{ and } {\overline{d}}_{H, \dots }=\left({d}_{H, \dots }^{1}+{d}_{H, \dots }^{2}\right)/2\end{array}$$

This procedure was chosen for two different reasons: First, for small eccentricities, the horizontal PSA is mainly determined by the effect of the pupil diameter on the horizontal calibration offset (${c}_{ H, \dots }^{2}-{c}_{ H, \dots }^{1}$) and not by the effect on the calibration gain (${d}_{ H, \dots }^{2}-{d}_{ H, \dots }^{1}$, see Results, Fig. 3a/b). Therefore, we ignored the effect of pupil diameter on the horizontal regression slope by using in Eq. (7) only a single slope (${s}_{H, \dots }^{*})$ defined as the inverse of the average calibration gain $(1/{\overline{d}}_{H, \cdots }$). Second, in agreement with previous studies (Ohlendorf et al., 2022), we assumed that the ratio ${s}_{V}/{s}_{H}$ is (for small eccentricities) identical to the ratio between the vertical and horizontal radius of the eye rotation (see Supplementary material for the derivation). For the human eye, this ratio is typically 0.76 (Ohlendorf et al., 2022). A control experiment was performed to verify that estimating ${s}_{V}$ from ${s}_{H}$ according to Eq. (7) is unbiased with a standard deviation of only 7% of its mean. In the following, we omit the subscripts *H* or *V* in all expressions that hold for both horizontal and vertical components.

#### PSA estimation and correction with the interpolation method

Pupil artifact correction was performed as follows. The calibrated left or right eye position ${e}_{\cdots }(t)$ for any given pupil position ${p}_{\cdots }(t)$ and pupil diameter pd(t) recorded during the following fixation paradigm was computed by interpolating between the two eye positions (Eq. 4):

8 $${e}_{\cdots }\left(t\right)=\left(1-\frac{pd\left(t\right)-{\overline{pd}}^{ 1}}{{\overline{pd}}^{ 2}-{\overline{pd}}^{ 1}}\right)\bullet {e}_{\cdots }^{1}(\mathrm{t})+ \frac{pd\left(t\right)-{\overline{pd}}^{ 1}}{{\overline{pd}}^{ 2}-{\overline{pd}}^{ 1}}\bullet {e}_{\cdots }^{2}(t)$$

where ${\overline{pd}}^{ i}$ denotes the mean pupil diameter observed during the calibration paradigm, averaged across the last 500 ms of all fixation periods at background luminance i. $pd\left(t\right)$ denotes the pupil diameter at the time of the actual raw pupil position data ${p}_{H, \cdots }(t)$ to be calibrated. Since the pupil constriction and dilation are binocularly synchronized in healthy controls, $pd\left(t\right)$ was defined by the pupil diameter of the left eye. All required calibration parameters (${o}_{\dots }^{i}$, ${s}_{\dots }^{i}$, ${\overline{pd}}^{ i}$) were collected specifically for each participant. For the bright and dark background luminance, the mean pupil diameter was ${\overline{pd}}^{ 1}$= 3.1 ± 0.3 mm and ${\overline{pd}}^{ 2}$= 4.4 ± 0.50 mm (*N* = 14), respectively. For the interpolation method to be applied to the subsequent fixation paradigm, this area must correspond to the pupil diameters occurring there. This was the case: $\text{3.2 mm}<pd<4.3 mm$.

The horizontal or vertical PSA was defined by the artifactual gaze shift [deg] that is corrected by the respective method. Thus, the interpolation method estimates the PSA as the negative of the compensatory gaze shift ${\Delta e}_{\cdots }$, i.e., the effect of a pupil dilation from ${\overline{pd}}^{ 1}$ to ${\overline{pd}}^{ 2}$ on the eye position (${e}_{\cdots }\left(t\right)$) for fixed pupil position ${p}_{\dots }={\overline{o}}_{\dots }:=\left({o}_{ \dots }^{1}+{o}_{ \dots }^{2}\right)/2$. Using Eq. (4), Eq. (5), and Eq. (8), the PSA results in

9 $${PSA}_{\dots }^{ip}=-{\Delta e}_{\cdots }={\left.{e}_{\cdots }^{1}\right|}_{{p}_{\dots }={\overline{o}}_{\dots }}-{\left.{e}_{\cdots }^{2}\right|}_{{p}_{\dots }={\overline{o}}_{\dots }}=\left({o}_{\dots }^{2}-{o}_{\dots }^{1}\right) {\overline{d}}_{\dots }$$

with ${\overline{d}}_{\dots }=\left({d}_{\dots }^{1}+{d}_{\dots }^{2}\right)/2$. If the calibration slopes do not depend on the pupil diameter, i.e., $\overline{d}={d}_{\dots }^{1}={d}_{\dots }^{2}$, then the PSA is equal to the difference between the calibration offsets (${PSA}_{\dots }={c}_{\dots }^{1}-{c}_{\dots }^{2}$, see Eq. 5).

The horizontal or vertical PSA gain estimated by the interpolation method (${G}_{PSA}^{ip}$) was defined by the artifactual gaze shift per pupil dilation $\Delta \overline{pd}={\overline{pd}}^{ 2}-{\overline{pd}}^{ 1}$, specified in units of [deg/mm]:

10 $${G}_{PSA}^{ip}=\frac{{PSA}_{\dots }^{ip}}{\Delta \overline{pd}}$$

The precision of this PSA gain estimate ${G}_{PSA}^{ip}$ was assessed by its within-subject standard deviation determined by the following error calculation. The horizontal and vertical PSA gains ${G}_{PSA}^{ip}$ of each eye are determined by *N* = 96 fixations quantified by *N* fixed horizontal target positions and N noisy measurement triples consisting of horizontal and vertical pupil position and pupil diameter. These measurement triples, namely [${p}_{H, \dots }^{1}\left(n\right) , {p}_{V, \dots }^{1}\left(n\right) , {pd}^{1}(n)$] and [${p}_{H, \dots }^{2}\left(n\right) , {p}_{V, \dots }^{2}\left(n\right) , {pd}^{2}(n)$] for$1\le n\le 48$, were all arranged in a large measurement vector $\underline{y}$ with 288 (= 3 × 96) elements. As the parameter vector $\underline{pv}={\left[{o}_{ \mathrm{H}, \dots }^{1}, {o}_{ \mathrm{H}, \dots }^{2}, {s}_{ \mathrm{H},\dots }^{1}, {s}_{ \mathrm{H},\dots }^{2}, {o}_{V, \dots }^{1}, {o}_{V, \dots }^{2}, {\overline{pd}}^{ 1}, {\overline{pd}}^{ 2}\right]}^{T}$ is derived from a linear regression of the measurement vector $\underline{y}$ on the target positions, $\underline{pv}$ is a linear function of $\underline{y}$ ($\underline{pv}=\mathbf{A} \underline{y}$). Consequently, the variance/covariance matrix (${{\boldsymbol{\Sigma}}}_{\underline{y}}$) of $\underline{y}$ can be estimated from the residuals of these regressions. The variance/covariance matrix of $\underline{pv}$ was then calculated as ${{\boldsymbol{\Sigma}}}_{\underline{pv}}=\mathbf{A} {{\boldsymbol{\Sigma}}}_{\underline{y}} {\mathbf{A}}^{T}$. Finally, small changes of the nonlinear function (${G}_{PSA}^{ip}=f(\underline{pv})$, Eqs. 5, 7, 9, 10) were linearized by $\Delta {G}_{PSA}^{ip}={\left({\left.\frac{d f(\underset{\_}{x)}}{d \underline{x}}\right|}_{x=\underline{pv}}\right)}^{T}\Delta \underline{pv}$, and the standard deviation of ${G}_{PSA}^{ip}$ was calculated by

11 $$SD\left({G}_{PSA}^{ip}\right)=\sqrt{{\left({\left.\frac{d f(\underset{\_}{x)}}{d \underline{x}}\right|}_{x=\underline{pv}}\right)}^{T} {{\boldsymbol{\Sigma}}}_{\underline{pv}} \left({\left.\frac{d f(\underset{\_}{x)}}{d \underline{x}}\right|}_{x=\underline{pv}}\right)}$$

#### PSA estimation and correction with the regression method

The regression method does not require manipulating pupil size during the calibration procedure. The PSA was ignored during calibration, and gaze position was calculated by using the average of the two calibration functions from Eq. (4):

12 $${e}_{\cdots }\left(t\right)=\frac{{e}_{\cdots }^{1}(\mathrm{t})+{e}_{\cdots }^{2}(t)}{2}$$

In the original study of Choe et al. (2016), the analysis was restricted to selected intervals, excluding blinks. Using a similar selection process, 76 fixation intervals, averaging 1.6 s, were selected and analyzed for each subject. The method of (Choe et al., 2016) was modified in that only the slow movement components (after exclusion of saccades and microsaccades) were, submitted to the subsequent regression analysis. Excluding saccades before regression should increase the method’s precision, as saccades superimpose the PSA without directly relating to pupil diameter. Thus, excluding them reduces noise in determining the regression slope, thereby approximating the PSA as a function of pupil diameter. The Supplementary material (section “Extraction of the slow-phase components of the eye position”) describes the selection of fixation intervals and the exclusion of microsaccades from the horizontal and vertical eye position (Eq. 12) in detail. Since this procedure also excluded all frequency components above 15 Hz (= 40 dB cutoff frequency), its output is called *slow-phase eye position* (*spe*). The purpose of this filtering is to optimize the precision of the estimated PSA gain by removing components of the eye position signal unrelated to changes in pupil diameter, such as the high-frequency noise of the pVOG signal (see Discussion). In a subsequent step, low-frequency components below 1 Hz were removed from the *slow-phase eye position* to focus on fixation drift within short time intervals. This is particularly important for the head-fixed pupil tracker used in the current study, which measures eye position in head-fixed coordinates. During fixation of a space-fixed target, slow head movements cannot be avoided across longer measurement periods. These head movements induce compensatory eye movements, which would impair the precision of the PSA gain estimate if they were not eliminated by the high-pass filter. The high-pass filter was implemented in the frequency domain (see Supplementary material, section “Spectral analysis”). The result of this preprocessing was a *filtered eye position* (*fe*) that did not contain saccades or frequency components below 1 Hz or above 15 Hz.

The same filtering was also applied to the pupil diameter (*pd*), to obtain the corresponding *filtered pupil diameter* (*fpd*) under exclusion of frequency components below 1 Hz or above 15 Hz.

The PSA gain was defined as the slope of a linear regression of the filtered eye position on the pupil diameter, pooled across all selected fixation intervals. The regression slope in the interval *k* was calculated by

13 $${\beta}_{k}=\frac{cov\left({\underline{fe}}_{k}, {\underline{fpd}}_{k}\right)}{var\left({\underline{fpd}}_{k}\right)}$$

where ${\underline{fpe}}_{k}$ and ${\underline{fpd}}_{k}$ denote the vectors containing all samples of the filtered eye position and filtered pupil diameter in the interval *k*. The regression slope ${\beta}_{k}$ is specified in units of deg/mm. The horizontal and vertical PSA gain was defined by the average of the respective regression slope (in deg/mm) across all *K* selected fixation intervals:

14 $${G}_{PSA}^{reg}=\frac{1}{K}\sum_{k=1}^{K}{\beta}_{k}$$

As for the interpolation method, the *precision of PSA gain estimate* was defined as the inverse within-subject standard deviation of the PSA gain estimate. Based on Eq. (14), this standard deviation was calculated as the standard error of the mean:

15 $$SD\left({G}_{PSA}^{reg}\right)=\sqrt{\frac{1}{K}} \sqrt{\frac{1}{K-1}\sum_{k=1}^{K}{\left({\beta}_{k}-{G}_{PSA}^{reg}\right)}^{2}}$$

PSA correction was achieved by multiplying the estimated PSA gain by the alternating components of the pupil diameter and subtracting this product from the original, uncorrected eye position (e(t), Eq. 12). The alternating components of pupil diameter were calculated by applying a high-pass filter to the pupil diameter pd(t). This high-pass filter was implemented by subtracting a low-pass filtered pupil diameter from the unfiltered pupil diameter. The finite impulse response of the low-pass was symmetric Gaussian with a standard deviation of 2 s.

#### Pupil position relative to the center of the limbus

To replicate the measurements of Wyatt (1995) concerning the pupil position relative to the limbus center, both the position of the center of the pupil and of the limbus were detected in the video streams of the left eye recorded in Experiment 2. Only video images from a subsection within 300 to 1200 ms of the target intervals (duration 1500 ms) were selected, in which the target was presented in the center of the screen and seen under monocular viewing conditions by the left eye. Half of these images were taken at the low background luminance (10 cd/m^2^) and half at the high background luminance (200 cd/m^2^). Images around the time of eye blinks in which the eye was not fully opened were excluded from the analysis. After this selection, 3128 images remained to be analyzed per subject. Each of the images was analyzed independently of all others. A deep learning algorithm was used to train a fully convolutional neural network for the segmentation of the pupil area (Yiu et al., 2019). This algorithm was further developed to segment the area of the cornea. The limbus was defined by the outer margin of the cornea. The limbus was manually edited using a dynamic mask editor to ensure that only visible parts of the limbus, i.e., those not hidden by the eyelids, were used for further processing (blue-marked pixels in Fig. 2).

**Fig. 2:**
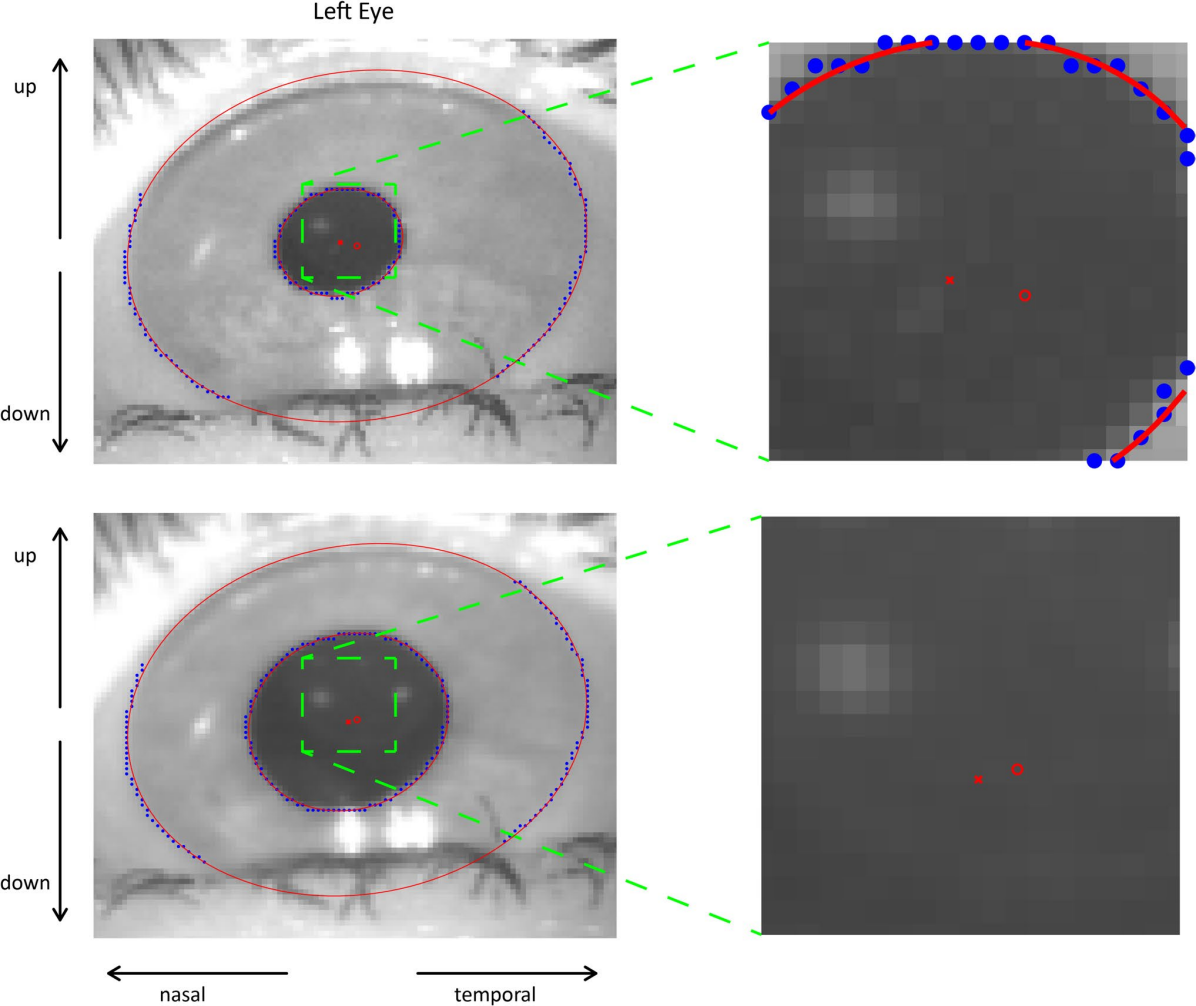
Evaluation of the pupil center relative to the limbus center. *Blue dots*: pixels that were identified as part of the limbus or part of the pupillary margin. *Red lines*: elliptical least-squares fit of the *blue dots*. *Red open circle*: center of the limbus ellipse. *Red cross*: center of the pupil ellipse. *Top*: Fitted pupil diameter: 3.08 mm; position of pupil center relative to limbus center: [horizontal, vertical] = [0.41, 0.08] mm; *Bottom*: fitted pupil diameter: 4.92 mm; position of pupil center relative to limbus center: [0.21, 0.05] mm. The horizontal shift of the red cross (0.2 mm) with pupil dilation indicates a shift in the temporal direction (towards the limbus center)

An ellipse was fitted to each of the pupil margin and the visible limbus. The sum of the squared geometric distances of the detected edge points to the respective ellipse was minimized. Compared to standard ellipse-fitting methods that minimize the algebraic distance (Gander et al., 1994), minimizing the geometric distance is computationally more time-consuming. However, if larger portions of the ellipse are concealed, as is usually the case with the limbus, minimizing the squared geometric distance yields more accurate results (Gander et al., 1994). The center of the fitted ellipses defined the center of the pupil and the center of the limbus. The relative pupil positions were defined as positive for pupil positions to the right (as seen from the participant) and upward from the limbus center. For each participant, the relative pupil positions were averaged over all images with low or high background intensity (corresponding to large or small pupil). The PSA in limbus coordinates (${PSA}^{Limbus}$) was defined by the difference in relative pupil position (dilated - constricted), and the corresponding artifact gain in limbus coordinates (${G}_{PSA}^{Limbus}$) was defined by

16 $${G}_{PSA}^{Limbus}=\frac{{PSA}^{Limbus}}{\Delta \overline{pd}}$$

where $\Delta \overline{pd}$ (dilated – constricted) denotes the difference in mean pupil diameter between the two luminance levels. The PSA gain in limbus coordinates (Eq. 16) was compared with the PSA gain in camera coordinates (${G}_{PSA}^{reg}$, Eq. 14) estimated from the same recording of the calibration paradigm of Experiment 2 with the regression method.

### Statistics

The confidence limits ${cl}_{95\%}$ of the mean (Figs. 5, 6, 10) were calculated as ${cl}_{95\%}=mean\pm SD/{N}^{0.5} tinv(1-0.05/2, N-1)$, where tinv denotes the inverse cumulative student-density distribution and *N* = 14 the number of participants. Effects with false positive probabilities less than 0.05 were considered significant.

#### PSA gain

Due to the specific symmetries of the estimated PSA gains, their magnitudes were defined as ($-{G}_{PSA, H,left}, {G}_{PSA, H,right}, {-G}_{PSA, V,leftt},{-G}_{PSA, V,right}$) and, submitted to a repeated measures ANOVA with the factors *direction* (horizontal/vertical), *eye* (left/right), and *estimation approach* (I/II/III).

#### Precision of the PSA gain estimate

To compare the non-normally distributed within-subject standard deviations of the PSA gain across direction, eye, and estimation method, the data were transformed using an exponential saturation function (y = $1-\mathrm{e}\mathrm{x}\mathrm{p}(-x/0.2$)) prior to statistical analysis. The normality of the transformed data was confirmed with the Lilliefors test before being analyzed. The within-subject standard deviation was compared between the methods by a repeated measures ANOVA (on the transformed data) with the factors *direction* (horizontal/vertical), *eye* (left/right), and *estimation approach* (I/II). Differences in the regression approach between the two recordings were examined using a repeated measures ANOVA with the same factors but with the estimation approaches (II/III) as levels of the third factor. Descriptive statistics are reported and presented in Fig. 6 as central values and confidence limits of the central values, calculated by back transformation of the mean and the confidence limits of the transformed data through inverse transformation ($x=-0.2*\mathrm{l}\mathrm{o}\mathrm{g}(y-1)$). Confidence intervals are reported as the difference between the confidence limits.

#### The coefficient of determination

The statistical analysis (ANOVA and post hoc tests) of hypotheses about the mean coefficient of determination (${\overline{R}}^{2}$) between the *filtered pupil diameter* and the *filtered eye position* was performed by transforming ${\overline{R}}^{2}$ using a power-logit function ($y=log\left({x}^{0.2}/\left(1-{x}^{0.2}\right)\right)$). The normality of the transformed data was also confirmed by the Lilliefors test. Descriptive statistics of ${\overline{R}}^{2}$ are reported as median [interquartile range]. The upper and lower limits of the 95% confidence interval of the median of ${\overline{R}}^{2}$ in Fig. 8 were calculated by an approximation of the distribution of the median ${\overline{R}}^{2}$ by bootstrapping. From the 14 measured ${\overline{R}}^{2}$ values, 10,000 data sets (*N* = 14) were generated by resampling with replacement.

## Results

### Comparison between the interpolation method and the regression method

#### PSA gain estimate

The estimation of the PSA gain by the interpolation method is illustrated for one representative participant in Fig. 3. The PSA was particularly evident in the dependence of the calibration offset on the pupil diameter. In contrast, the calibration gain was relatively insensitive to changes in pupil diameter.

**Fig. 3:**
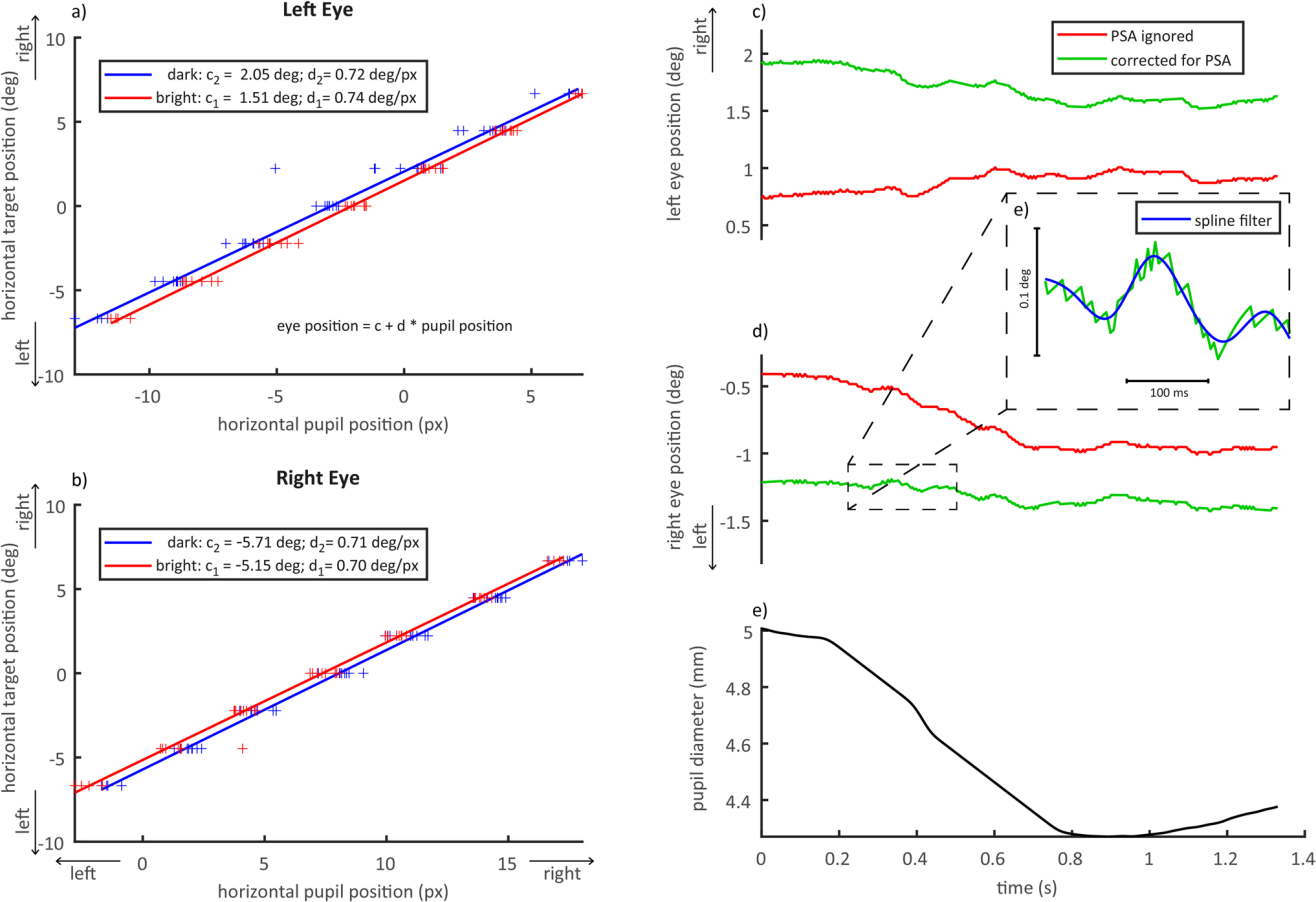
PSA correction with the interpolation method in a representative participant. The horizontal calibration curves for the left **a)** and right **b)** eye shift with pupil dilation (*blue: dark background*) in the temporal direction, i.e., rightward for the right eye and leftward for the left eye. During a single fixation interval of the subsequent fixation paradigm, the unfiltered position of the left **c)** and right **d)** eye is shown before (*red*) and after (*green*) the PSA correction and extraction of the slow-phase component. The inset **e)** shows in blue the smoothing of the corrected data achieved by the spline filter. The spontaneous constriction of pupil diameter by 0.7 mm **e)** induced a convergent PSA (*red* in c, d), which is corrected by the interpolation method (*green* in c, d). Note that the extent of this correction is in the order of magnitude of the actual fixation drift

This is demonstrated by the fact that the two calibration lines for dark and light backgrounds do not intersect within the range of target eccentricities presented (Fig. 3a/b). For the left eye (Fig. 3a), the calibration offset was greater for the dark background (large pupil diameter) than for a bright background (small pupil diameter), whereas this effect was reversed for the right eye (Fig. 3b). On average across participants, pupil dilation during the calibration paradigm caused a PSA in the temporal direction in both eyes, i.e., leftward for the left eye (${PSA}_{H, left}^{ip}$=—0.28 ± 0.20 deg; T(13) = 5.4; *p* < 0.001) and rightward for the right eye (${PSA}_{H, right}^{ip}$= 0.35 ± 0.17 deg; T(13) = 7.7; *p* < 0.0001). The horizontal PSA was left/right antisymmetric, as the paired sum of the PSA of both eyes did not significantly differ from zero (T(13) = 1.00; *p* = 0.33). Changes of pupil diameter varied between participants, the two paradigms, and the data used by the different approaches to quantify the PSA. To compare the influence of pupil diameter on apparent eye position between participants, paradigms, and estimation approaches, the PSA gain was used. This gain factor was defined as the change in apparent eye position (deg; positive towards the right and upwards) per increase in pupil diameter (mm). Figure 4 shows how the PSA gain was estimated from fixation-paradigm data using the regression method (Estimation Approach III). Although the spontaneous changes of both pupil diameter and apparent eye position (0.1 deg) were small (less than 0.1 mm and 0.1 deg, respectively), the quality of the pVOG signal was good enough to detect the dependency between the two signals.

**Fig. 4:**
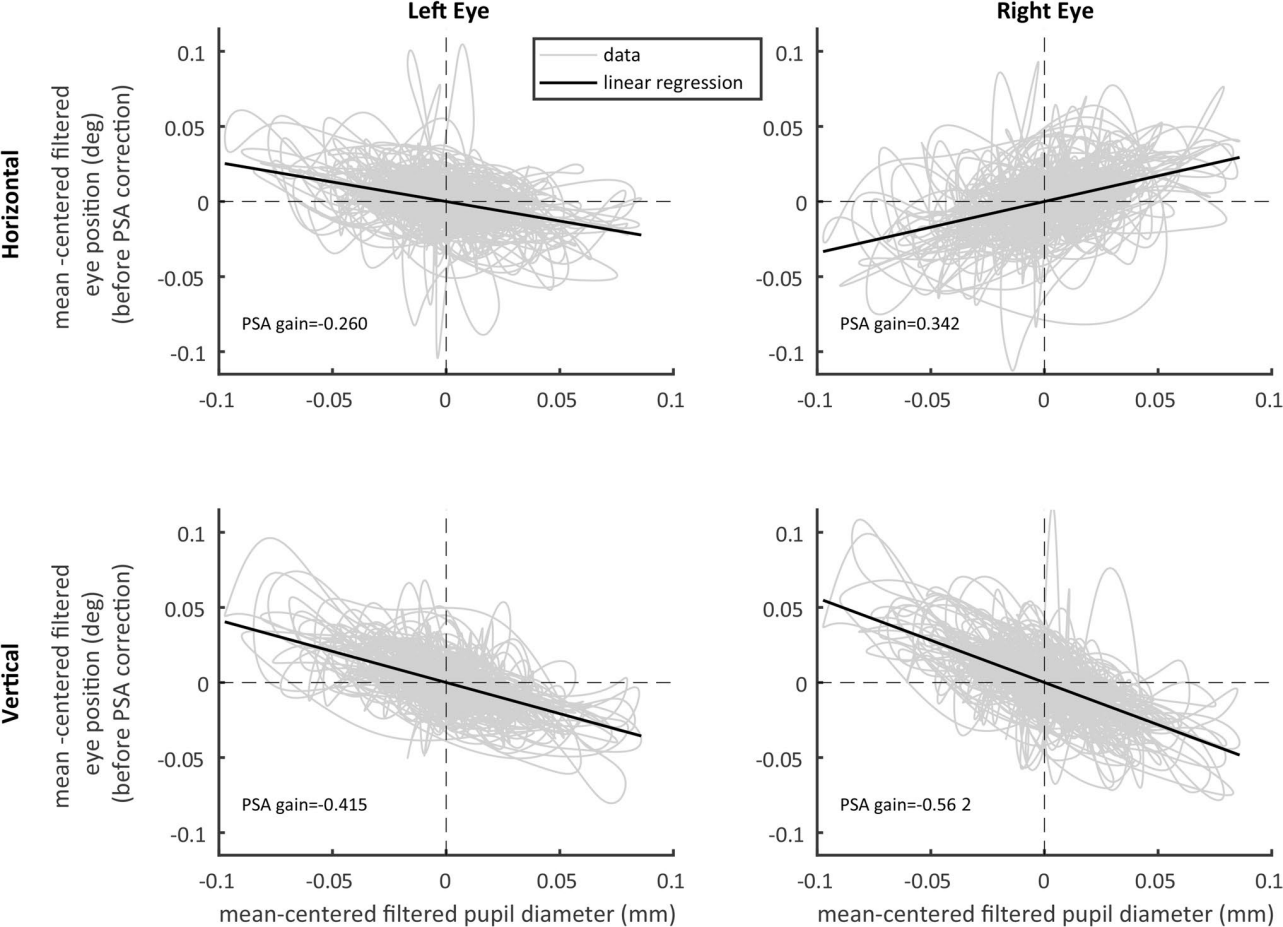
*Thin gray lines*: Spontaneous changes in apparent eye position and pupil diameter of a single participant in the selected intervals of the fixation interval. Microsaccades and frequency components below 1 Hz or above 15 Hz were removed to improve the precision of the PSA gain estimate. *Solid black lines*: Linear regression of filtered eye position on filtered pupil position. *Dashed lines*: Zero reference lines indicating that pupil diameter and eye position were mean-centered within each fixation interval. The regression method (estimation approach III) quantified the PSA gain by slope of this regression line

Figure 5 shows the PSA gain for horizontal and vertical directions, both eyes, and the three different estimation approaches (I, II, III). None of the confidence intervals included zero, indicating that the PSA gain was significant in both the horizontal and vertical directions. Pupil dilation resulted in a downward and temporal displacement of the pupil. Submitting the magnitude of the PSA gain to a repeated measure ANOVA with the factors *direction* (horizontal/vertical), *eye* (right/left), and *estimation approach* (I/II/III) showed a highly significant main effect of the factor *direction* (*F*(1,13) = 36.1; *p* < 1e-4) and no other significant (*p* > 0.25) main or interaction effect. Thus, the different estimation approaches did not differ systematically. The results also confirmed that the mean horizontal PSA gain was antisymmetric left/right and further showed that the mean vertical PSA gain was symmetric left/right. The mean horizontal PSA gain magnitude $\left({G}_{PSA, H,right}-{G}_{PSA, H,left}\right)/2$), averaged across all methods and participants, was 0.23 ± 0.09 deg/mm and was smaller than the mean vertical PSA gain magnitude $-\left({G}_{PSA, V,right}+{G}_{PSA, V,left}\right)/2$ = 0.62 ± 0.21 deg/mm. For the vertical direction, the left/right symmetry also holds for most individuals, as Pearson’s coefficient of correlation between left and right vertical PSA gain (*r* = 0.61) was significant (T(12) = 2.69; *p* = 0.02). The correlation between left and right horizontal PSA gain (*r* = – 0.09) did not reach significance (T(12) = – 0.32; *p* = 0.76), indicating that some individuals deviated from the typical left/right antisymmetric pattern.

**Fig. 5:**
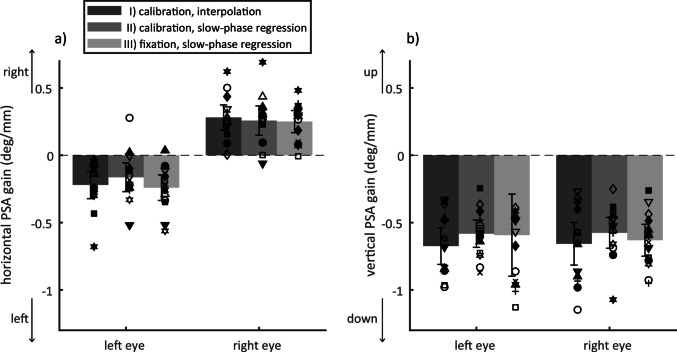
PSA gain evaluated with the different estimation approaches (I, II, III, see Objectives), eyes (left, right), and directions [horizontal: **a)**; vertical: **b)**]. The gain values are expressed as an artifactual change in eye position in deg per mm of pupil dilation. *Symbols* denote individuals, *bars* denote the mean values. *Whiskers* indicate the 95% confidence interval of the mean. Pupil dilation caused artifacts in the temporal and downward directions. The magnitude of vertical PSA gains was larger than that of the horizontal PSA gains. The PSA gain did not differ between the estimation approaches

With the interpolation method (estimation approach I), the standard deviation of the pupil diameter across all selected fixations was 0.75 mm (RMS, *N* = 14). In the regression method, the standard deviation of the pupil diameter was evaluated under exclusion of frequency components below 1 Hz (Eq. 20). In the calibration paradigm (estimation approach II), it was 0.14 mm. When analyzing the fixation paradigm (estimation approach III), the standard deviation of the pupil diameter was only 0.02 mm. It was the largest for the interpolation method because, in the calibration paradigm, the largest changes in pupil diameter occurred between, not within, the fixation intervals selected by the regression method (estimation approach I). Within the fixation intervals selected by the regression method, changes in pupil diameter were larger in the calibration paradigm (estimation approach II) than in the fixation paradigm (estimation approach III), possibly due to differences in the task and to the background luminance that changed during the calibration paradigm but not during the fixation paradigm.

#### Precision of PSA gain estimates

Figure 6 shows the inverse precision of the PSA gain estimate, defined as the within-subject standard deviation of the PSA gain estimates. The comparison between the two estimation approaches of the interpolation method and the regression method was examined with a repeated measures ANOVA with the factors *direction* (horizontal/vertical), *eye* (left/right), and *estimation approach* (I/II). None of the main effects or interaction effects reached significance (p > 0.11). Thus, the precision of PSA gain estimate did not differ between the two methods, the directions, or the two eyes.

**Fig. 6:**
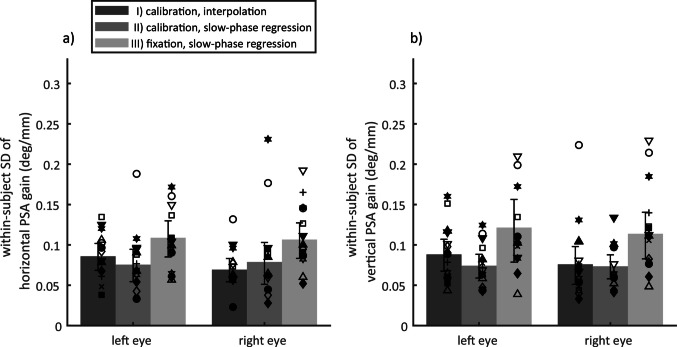
Within subject-standard deviations the PSA gain estimated with the different estimation approaches (I, II, III, see Objectives), eyes (left, right), and directions [horizontal: **a)**; vertical: **b)**]. *Symbols* denote individuals, *bars* the central values. *Whiskers* indicate the 95% confidence interval of the central values. The standard deviations did not differ systematically between the estimates of the regression or interpolation methods (I/II). Variable errors of the regression method were larger in the fixation paradigm than in the calibration paradigm (II/III)

The comparison of the precision of the PSA gain estimates of the regression approach between the calibration and fixation paradigm (II/III) showed larger within-subjects standard deviation in the fixation paradigm (0.113 [0.050] deg/mm) than in the calibration paradigm (0.076 [0.034] deg/mm; main effect factor *estimation approach*: *F*(1,13) = 20.82; *p* < 1e-3). As in the last paragraph, the precision of the PSA gain estimate did not differ between the two directions or between both eyes since no other main effect or interaction of the factors *direction* (horizontal/vertical), *eye* (left/right), or *estimation approach* (I/II) reached significance (*p* > 0.26).

The overall mean of the within-subject standard deviation across directions, eyes, and estimation approaches was 0.09 [0.04] deg/mm, constituting a considerable fraction of the horizontal (39%) or vertical (15%) PSA gain.

#### Stability of the PSA gain over time

As shown in the section “PSA gain estimate”, the PSA gain did not differ significantly between the calibration and fixation paradigms (bars II/III in Fig. 5). The individual paired difference in the PSA gain (symbols II/III in Fig. 5) between the two recordings (${\Delta G}_{PSA}^{reg}$) was distributed around zero across participants. It therefore remained unclear whether these individual differences between the two recordings reflected genuine temporal variability in PSA gain or were attributable to noise of the PSA gain estimate. To dissociate these possibilities, we computed non-centered z scores of ${\Delta G}_{PSA}^{reg}$ for each participant, direction, and eye. Each *z*-score was obtained by normalizing ${\Delta G}_{PSA}^{reg}$ (symbols II/III in Fig. 5) by the root sum of squares of the corresponding within-subject standard deviations (symbols II/III in Fig. 6). The null hypothesis that all PSA gains are stable is equivalent to the hypothesis that the expected value of each individual ${\Delta G}_{PSA}^{reg}$ is zero. In this case, both the distribution of centralized and the non-centralized z-scores are identical to the standard normal distribution shown on the left in Fig. 7. The mean square of the non-centralized *z*-score was 2.10, which was significantly (chi2(56) = 117.6; *p* < 0.0001) greater than one, i.e., greater than the mean square of the standard normal distribution. Therefore, the stability assumption did not hold for all cases. This is also shown by the fact that the 13 cases outside the 95% confidence interval (solid symbols in Fig. 7) accounted for 23% of the total number, which was significantly higher than the 5% expected under the null hypothesis.

**Fig. 7:**
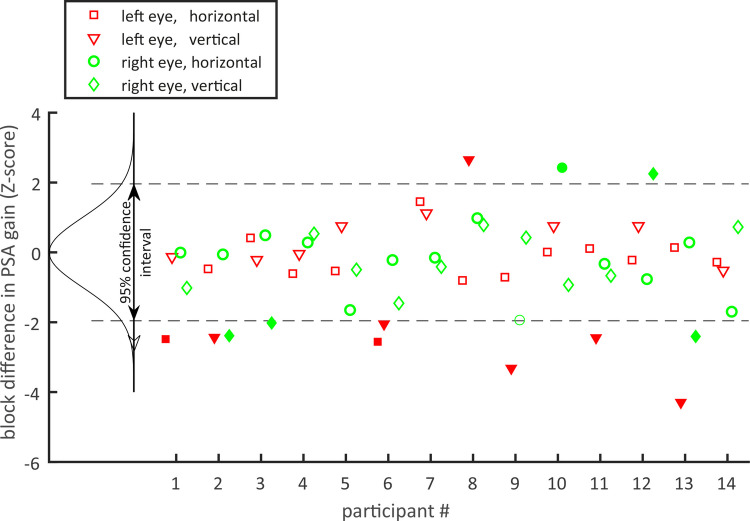
*Symbols*: Non-centralized z-scores of the PSA gain difference (${\Delta G}_{PSA}^{reg}$) between the fixation paradigm and the calibration paradigm, as estimated with the regression method (estimation approaches II/III). *Black solid*: Standard normal distribution with mean zero and standard deviation one. *Dashed*: 95% confidence interval at *Z* =  ± 1.96. Open symbols indicate non-significant differences within the 95% confidence interval. *Closed symbols*: Significant differences. Twelve of 56 differences (23%) differed significantly from zero

In summary, in some participants, horizontal or vertical PSA gains showed temporal instabilities within a measurement interval of about 12 min, even though ${\Delta G}_{PSA}^{reg}$ did not differ significantly from zero in most cases (77%).

#### Effectiveness of PSA correction

The effectiveness of the PSA corrections was evaluated by the mean coefficient of determination (${\overline{R}}^{2}$) between pupil diameter and slow-phase eye position in the fixation paradigm. Before the correction (Fig. 8a), ${\overline{R}}^{2}$ was significantly (main effect *direction*: *F*(1,13) = 29.54; *p* < 2e-4) larger in the vertical (median [interquartile range] = 14% [12%]) than in the horizontal direction (1.5% [2.6%]). No other effect or interaction of the repeated measures ANOVA with the factors *direction* (horizontal/vertical) and *eye* (left/right) reached significance (*p* > 0.5). The large difference between the two directions corresponds to the direction effect of the PSA gain magnitude (Fig. 5a/b).

**Fig. 8:**
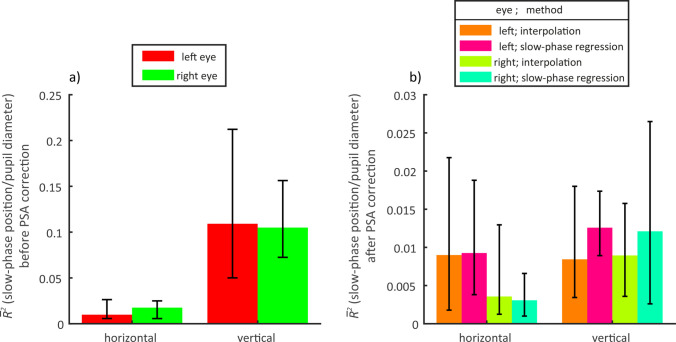
The mean coefficient of determination (${\overline{R}}^{2}$) indicating the fraction of the variance of slow-phase position explained by pupil diameter before **a)** and after **b)** correcting for PSA. *Bars*: median across participants. *Whiskers*: Upper and lower limits of the 95% confidence interval of the median. **a)** Pupil diameter could explain about 18% of uncorrected slow-phase position. **b)** The correction methods reduced these ${\overline{R}}^{2}$ values to 0.6% for the horizontal and to 1% for the vertical drift components.

To correct the data from the fixation paradigm, the interpolation method used the PSA gain estimate from the calibration paradigm (estimation approach I). For the regression method, the first half of the fixation paradigm was used to estimate the PSA gain, which was then used to correct the filtered eye position in the second half of the paradigm. The ${\overline{R}}^{2}$ values after correction are shown in (Fig. 8b). The repeated measures ANOVA with the factors *direction* (horizontal/vertical), *eye* (left/right), and *method* (interpolation/regression) did not show any significant main effect or interaction (*p* > 0.17) in particular, no significant effect involving the factor *method*. Thus, the effectiveness of both correction methods did not differ when PSA gain and the residual ${\overline{R}}^{2}$ were calculated in different data sets. Averaged across both eyes and methods, the correction reduced the ${\overline{R}}^{2}$ of the horizontal drift components to 0.6%, and that of the vertical drift components to 1%.

When the same data set was used for both estimation of the PSA gain and the correction, the regression method effectively eliminated the coefficient of determination (${\overline{R}}^{2}$= 0.01% [0.02%]). However, this usage of the regression method would provide a benefit only if changes in the PSA gain occur between data sets. The stability analysis of the previous section showed that such changes occurred only in a minority of cases.

#### Spectral analysis of fixation drift

The power spectral density of the fixation drift was evaluated after applying PSA correction with the regression method because it outperformed the interpolation method. Figure 9 shows the power spectral density (PSD) of drift position in comparison to methods that do not measure pupil position and are therefore not subject to the PSA. Compared to the measurement with the search coil (Findlay, 1971) or the dual-Purkinje-image (DPI) technique (Ko et al., 2016), the measurement with the head-mounted pupil tracker of the current study (red solid line in Fig. 9) declines more rapidly for frequencies above 10 Hz. This is a byproduct of the low-pass filter with the cutoff frequency of 10 Hz (see Methods). However, for the frequency band of interest between 1 and 10 Hz, the horizontal fixation drift (Fig. 9a) of the current study showed PSD values between the two previous studies and shared with both the decline with frequency of 6 dB per octave. It has been questioned whether this decline reflects a characteristic of true fixation drift and to what extent it may be affected by the application of filters (Niehorster et al., 2021). The spline filter used in our study cannot explain this decline because its frequency response remains approximately constant below the 3-dB cutoff frequency (Fig. 9, green). The observed 6 dB/oct decline indicates that the PSD of fixation-drift velocity was constant across the considered frequency range (1–10 Hz). Most studies, including Findlay (1971), have not reported the PSD of vertical fixation drifts. In the current study, the PSD of the vertical drift (Fig. 9b) showed a similar frequency dependence as that of horizontal drift. In summary, Fig. 9 shows that the presented method has largely avoided distortion of the PSD in the frequency band of interest.

**Fig. 9:**
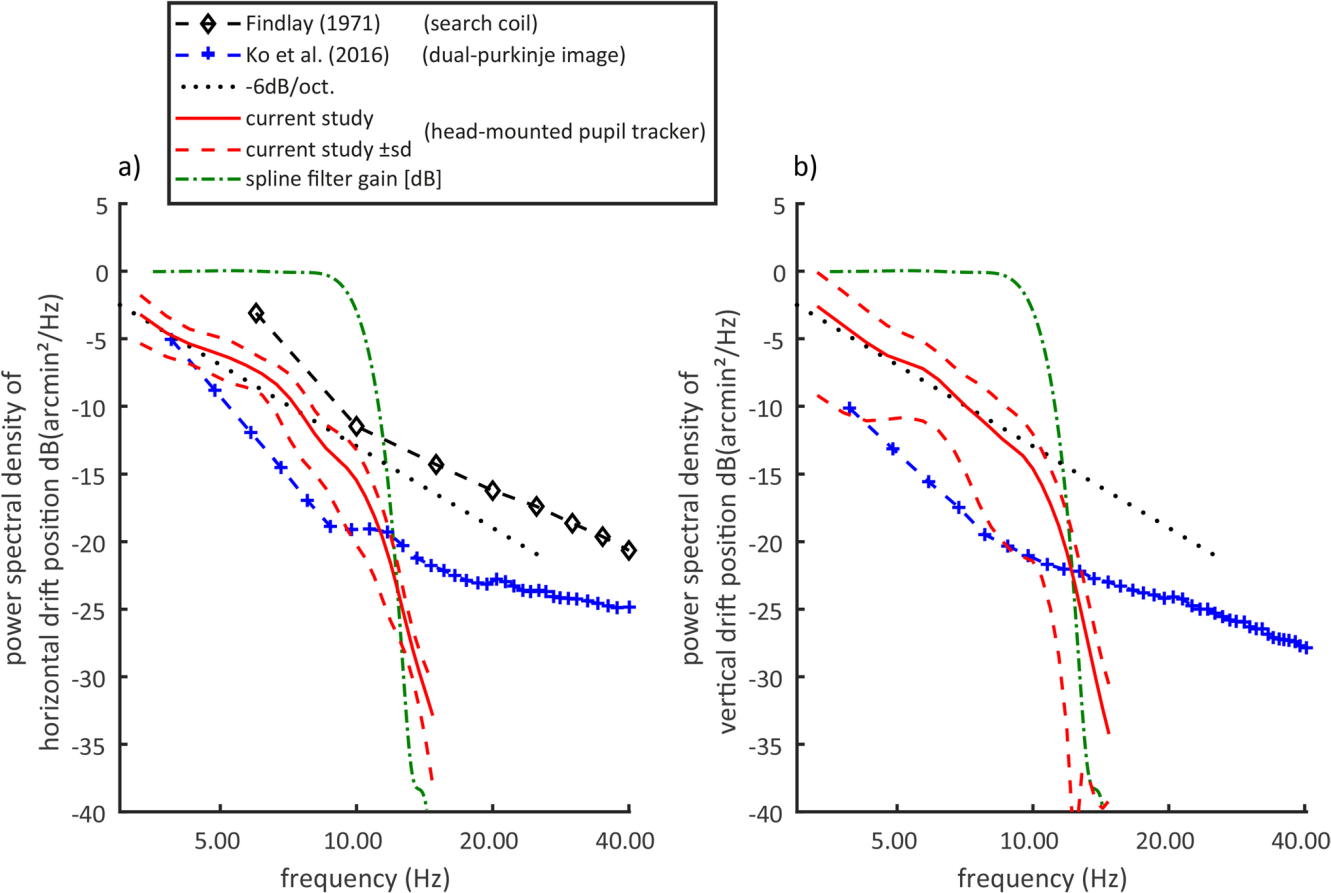
The power spectral density (PSD) of the horizontal **a)** and vertical **b)** drift position ($2 {PSD}_{drift}\left(f\right)$, Eqs. A11, A12) reported by different studies and recording systems. *Black dashed line*: Search coil technique (Findlay, 1971), one participant. This study did not quantify the PSD of vertical fixation drift. *Blue dashed line*: Dual-Purkinje-image technique (Ko et al., 2016), four participants. *Red solid line*: Head-mounted pupil tracker and the regression method of the current study, averaged across 14 participants. *Red dashed line*: Mean ± standard deviation of the PSD (*N* = 14). *Black dotted line*: Illustration of a decline of a PSD of drift position with 1/f.^2^ (= – 6 dB/oct.), which corresponds to a drift velocity with constant PSD. *Green dashed dotted line*: Gain of the spline filter (dB)

### Comparison between PSA in limbus coordinates and camera coordinates

The results of Experiment 2 were as follows. On average, the pupil center was displaced with respect to the limbus center in the nasal and upward direction and moved with dilation closer to the limbus center (in the temporal and downward direction, Fig. 10 a. A repeated measures ANOVA on PSA gains, with *direction* (horizontal/vertical) and *coordinates* (camera/limbus) as factors, showed that the magnitude of the vertical PSA gain was greater than that of the horizontal PSA gain (main effect *direction*: *F*(1,10) = 5.0; *p* = 0.049).

**Fig. 10:**
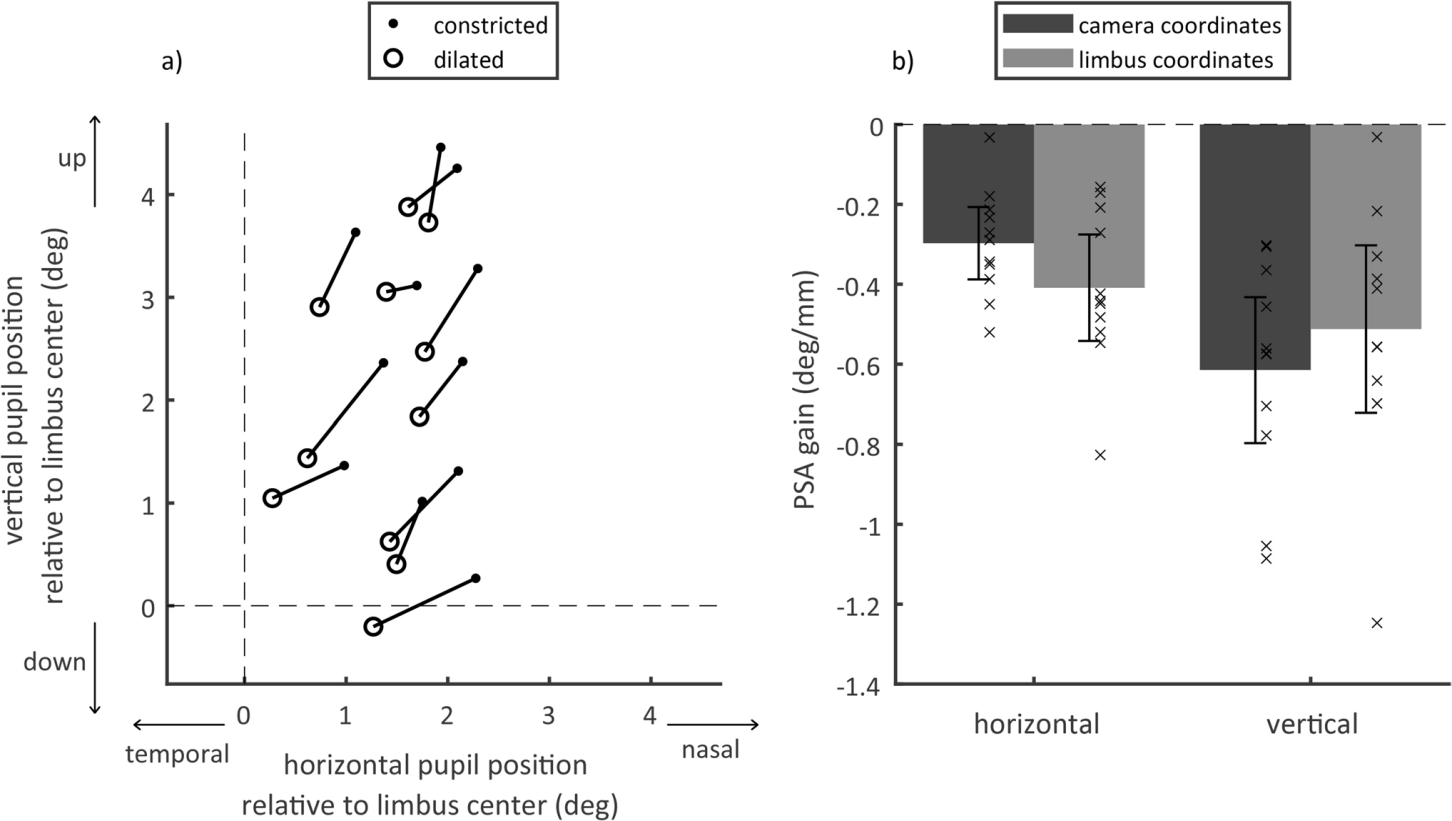
**a)** Pupil position of the left eye in limbus coordinates averaged across all selected video frames for constricted (*solid*) or dilated pupil (*open*). Each symbol shows an individual (*N* = 11). **b)** PSA gains evaluated in camera coordinates (${G}_{PSA}^{reg}$, Eq. 14) and in limbus coordinates (${G}_{PSA}^{Limbus}$, Eq. 16). Negative PSA gains indicate that there were artifactual pupil shifts to the left and down for pupil dilation. *Crosses*: individuals. *Bars*: mean across participants. *Whiskers* indicate the 95% confidence interval of the mean

The main effect and the interaction involving the factor *coordinates* did not reach significance (*p* > 0.08). These results confirmed the observations of Wyatt (1995) and further showed that the vectorial pupil shift relative to the limbus center and relative to the camera image did not differ significantly. This supports the hypothesis that the PSA under the conditions of Experiment 2 is caused mainly by movements of the pupil relative to the eye due to anisotropic constriction and dilation of the pupil.

## Discussion

Eye position measurements acquired with pVOG are confounded by the pupil-size artifact (PSA). Previous studies (Drewes et al., 2014; Hooge et al., 2021; Jaschinski, 2016; Wildenmann & Schaeffel, 2013; Wyatt, 1995) observed that pupil dilation caused a pupil shift in the temporal direction (i.e., opposite to the nasal direction). Confirming these results, the current study observed a group average of about 0.23 deg horizontal pupil shift per millimeter of pupil dilation with considerable between-subjects standard deviation. However, although the antisymmetric pattern of these shifts (to the left in the left eye and to the right in the right eye) held for the group average, this was not the case for each individual. The literature is less consistent about the direction of the vertical components of the PSA. For pupil dilation, downward PSA (Wildenmann & Schaeffel, 2013; Wyatt, 1995), upward PSA (Drewes et al., 2014), as well as vertical PSA shifts that depend nonlinearly on pupil diameter (Hooge et al., 2021) have been observed with pupil dilation. In our data, pupil dilation was associated with a downward PSA that was greater in magnitude (0.62 deg/mm) than the horizontal PSA (0.23 deg/mm), though it also showed a large between-subjects standard deviation. This illustrates the importance of determining the PSA individually for each person and each eye.

Experiment 1 also showed that, without PSA correction, pupil diameter accounted for approximately 14% of the variance of the vertical but only for 1.5% of the horizontal fixation drift (${\overline{R}}^{2}$, Fig. 8a). This shows that pVOG measurements of fixation drift, in particular its vertical components, are contaminated by PSA and can be significantly improved by successful PSA correction. Two offline correction methods were used, both of which aim to minimize the coefficient of determination (${\overline{R}}^{2}$) between pupil diameter and pupil position. An important justification for this aim is provided by Experiment 2, in which it was shown that the PSA could be explained by movements of the pupil relative to the eyeball. Conversely, this means that the orientation of the eyeball – and thus the direction of gaze – did not change with the pupil diameter. This confirms that the reduction of ${\overline{R}}^{2}$ is a suitable measure for the efficiency of the PSA correction methods. Both methods reduced the mean coefficient of determination (${\overline{R}}^{2}$) to 40% (horizontal) and to only 7% (vertical) of its value before correction (Fig. 8b). This demonstrates the ability of these methods to enhance pVOG measurements of fixation drift.

The analysis of the individual PSA gain estimates showed that they did not differ significantly between the methods (Fig. 5). The precision of the PSA gain estimates did not differ between the methods when applied to the same data (Fig. 6, bars I/II) but the regression method could estimate the PSA gain more precisely in the calibration paradigm than in the fixation paradigm (Fig. 6, bars II/III). The two methods differed mainly in that the interpolation method estimated and corrected the PSA gain from separate, successively recorded data sets, whereas the regression method used a single data set for both. This difference may represent a potential advantage of the regression method, as the stability analysis indicated that PSA gain differed significantly between the two datasets in 23% of all cases (Fig. 7).

Before discussing these results in more detail, we must consider some general issues related to measuring fixation drift, as well as some specific features of the pVOG device used.

### Advantages and disadvantages of a head-mounted pupil tracker for measuring fixation drift

For this study, we used a head-mounted pupil tracker (EyeSeeCam®, EyeSeeTec GmbH, Munich, Germany) that measures eye movements in head-fixed coordinates. Thereby, it differs from the pVOG used by Drewes et al. (2014) and (Choe et al., 2016), (EyeLink 1000®, SR Research, Ottawa, Canada), which measures eye position with respect to a remote camera fixed in the external space. Similar to these two studies, most research on PSA has employed pVOG systems that estimate gaze position in space by analyzing the difference vector between the pupil center and the corneal reflection of a fixed infrared light source (the PC-CR difference). In contrast, the head-mounted pupil tracker used in the current study determines eye position in head-centered coordinates by relying solely on the pupil center in head-fixed coordinates.

There are two reasons for this choice. First, head-fixed coordinates are of particular importance for the investigation of fixation drift, as these drifts are not significantly influenced by the eye position in relation to an external fixation target (Engbert & Kliegl, 2004). Thus, in contrast to visually guided eye movements, fixation drift is usually measured in head-fixed coordinates rather than in world-fixed coordinates. The second reason is that the pupil center directly corresponds to the presumed output variable of the PSA, whereas the PC-CR difference incorporates the corneal reflex as an additional, potentially noisy variable. The calibration of the raw signal of the head-mounted pupil tracker into eye position in head-fixed coordinates is also easier than that of the PC-CR difference into world-fixed coordinates.

The inherent disadvantage of head-fixed coordinates is that, without an extremely rigid mechanical stabilization, it is practically impossible to immobilize the head position over long periods of time. During fixation of a space-fixed target, involuntary head movements, especially with low-frequency components, are therefore unavoidable. These lead to visually induced tracking movements that compensate for the relative movement of the target to the head and are superimposed on the actual fixation drift. Without rigid mechanical stabilization, low-frequency components of the fixation drift can therefore not be measured correctly. This is why frequency components below 1 Hz were excluded from analysis.

A characteristic noise source in pVOG is related to the nominal sub-pixel resolution of the pupil position (0.01 deg for the EyeSeeCam®), achieved by integrating over the positions of many pixels on the pupil edge. This resolution is achieved only under optimal and constant illumination and full visibility of the pupil margin. Occasional impairment of signal quality manifested in the EyeSeeCam® in small artifactual signal oscillations above 15 Hz. This is why frequencies above 15 Hz are excluded in the current study. This choice of the upper frequency limit was rather conservative, as the EyeSeeCam® demonstrates good signal quality above 15 Hz under constant illumination with full visibility of the pupil margin.

While previous studies on PSA correction have not specified to which type of eye movements the correction should be applied, the present study focuses on fixation drifts. In particular, it focuses on those frequency components that can best be measured with the pupil tracker used.

### Generalizability to PC-CR systems

The current study was conducted using a head-mounted pupil tracker, as this provides a position signal that is most directly influenced by the PSA and is therefore ideal for investigating and quantifying the PSA. In contrast, in systems that measure eye position in world coordinates based on the PC-CR difference, only the pupil center is affected by the PSA, but not the corneal reflex. However, since these systems only use the difference between the two signals, the influence of the PSA on the PC-CR difference is the same as that on the pupil center (examined in the current study). Given that the methods presented in the current study only perform an additive correction of the pupil signal, it would be appropriate to apply the same correction to the PC-CR signal. In this study, we observed PSA gains of 0.23 deg/mm in the horizontal direction and 0.62 deg/mm in the vertical direction. These gains quantify the extent of the position error caused by changes in pupil diameter and most likely also generalize to PC-CR systems.

### Stability of the PSA over time

Hooge et al. (2021) observed the PSA in two participants in several repeated measures and concluded “… that the pupil-size artefact is very reproducible on a time scale shorter than 1 h”. This agrees with the current study insofar as the majority (77%) of our participants did not show a significant difference between the PSA gains of the two measurements obtained within only 12 min. However, due to the larger number of subjects (*N* = 14), the current study was able to show that the non-systematic PSA gain changes were, on average, too large to be explained exclusively by random estimation errors. Although this result suggests that temporal instabilities in PSA gain may occur, obvious violations of PSA gain stability were observed only relatively rarely.

### Absence of systematic differences in the estimated PSA gain between methods

In the calibration paradigm, the standard deviation of the pupil diameter within the analyzed data with the interpolation method (estimation approach I: 0.75 mm) was five times as large as with the regression method (estimation approach II: 0.14 mm). This constitutes an important difference between the two methods and would have led to differences in the PSA gain estimates if the PSA gain, expressed as a function of pupil diameter, were highly nonlinear. However, there was no systematic difference between these two PSA gain estimates (Fig. 5), suggesting that PSA gain is approximately linear with respect to pupil diameter, at least in the range of pupil diameters between 3.1 and 4.4 mm used in the calibration paradigm of the current study. In our fixation paradigm, the range of the pupil diameters during the fixation intervals was even smaller with a standard deviation of only 0.02 mm (Fig. 4). However, for changes in pupil diameter of more than 1.5 mm, a linear approximation of the PSA is likely insufficient, as under such conditions—particularly for the vertical PSA components—clear nonlinearities have been observed in previous studies (Culemann et al., 2026; Hooge et al., 2021).

### Comparing the precision of PSA gain estimates between methods

In both the interpolation and regression methods, the precision of the PSA gain estimation basically depends on the number of independent data pairs [pupil position, pupil diameter] used for estimation, and the ratio between the standard deviation of pupil diameter and the standard deviation of the components of the measured eye position signal unrelated to pupil parameter. When estimating PSA gain, these components play the role of noise. Consequently, three different aspects of the two estimation methods must be considered.

Firstly, regarding the number of data pairs, the regression method uses not just a single pair of data per fixation, as the interpolation method does, but all samples collected during a fixation period. This results in a larger number of data samples used for estimation and constitutes a potential advantage of the regression method. Secondly, the larger standard deviation of the pupil diameter in the interpolation method (estimation approach I) represents an advantage over the regression method (estimation approach III). Third, regarding the noise of the PSA gain estimate, it should be noted that the PSA estimate of the interpolation method is sensitive to low-frequency pVOG noise components of the pupil position. The head-mounted pupil tracker is particularly susceptible to such low-frequency interference as it can be caused by slow head movements (as discussed in the section “Advantages and disadvantages of a head-mounted pupil tracker …”). In contrast, the regression method depends less on low-frequency pVOG noise. This is partly achieved by re-centering of each fixation interval to zero, a procedure that was also applied by Choe et al. (2016). In addition, the regression method of the current study further reduced the effects of low frequencies by estimating the PSA gain using spectral analysis to ignore frequencies below 1 Hz. This filtering reduces the level of pVOG noise and improves the precision of the regression method compared to the interpolation method.

On the one hand, these different factors apparently compensated for each other since the precision of the PSA gain estimate did not differ between the two methods (Fig. 6, estimation approaches I/II). On the other hand, the precision was better in the calibration paradigm than in the fixation paradigm (Fig. 6, estimation approaches II/III). This difference is likely due to the larger standard deviation of pupil diameter in the calibration paradigm (0.14 mm) compared to the fixation paradigm (0.02 mm). This suggests that estimating the PSA gain from the calibration paradigm (with either method) is more precise than the estimation from the fixation paradigm. However, this conclusion holds only under the assumption that the actual PSA does not differ between the two recordings, which has been challenged by the present results concerning the stability of the PSA over time.

Overall, it is remarkable that the regression method could compensate for the PSA with similar precision as the interpolation method, even though it does not require any experimental modulation of pupil size, despite the simplicity of its underlying model, and despite its neglect of position dependencies and of nonlinearities of the PSA. It must be emphasized that the reported efficiency of the regression method depends critically on the specific experimental conditions of the current experiment, namely the limited range of eye position and the occurrence of only moderate changes in pupil diameter. A variety of topics concerning fixation drifts, e.g., their binocular coordination or their dependence on mental activity, can be investigated under these constraints. Of course, there are many other topics that are incompatible with the strict limitation on eye position. For example, the dependence of fixational drifts on eye position, which is of clinical interest in pathological deficits of gaze holding. For any experimental condition requiring a larger variety in eye position, the interpolation method may be the better choice.

### *Differences between the regression method of the current study and the approach of *Choe et al., 2016

The current study estimated the PSA gain by a regression of the slow-phase components of eye position on pupil diameter, excluding frequency components below 1 Hz. In contrast, Choe et al. (2016) used the original eye position, including microsaccades. This modification was done here to optimize the regression method for the measurement of fixation drift (frequency components > 1 Hz), whereas most previous studies aimed to improve the precision of fixation errors (frequency components < 1 Hz). Previous studies did not evaluate the precision of the PSA gain estimate. The current study showed that the coefficient of variation of the PSA gain estimate, i.e., the ratio between the within-subject standard deviation of the PSA gain estimate and its mean, was relatively large (horizontal: 39%; vertical: 15%). Thus, the precision of this estimate was not very high. Under these circumstances, it becomes crucial that the signal from which the PSA is estimated is as similar as possible to that to be compensated for the PSA. This is achieved by the modified regression method by estimating the PSA gain directly from the signal of interest, i.e., the slow-phase eye position during the fixation paradigm. The decorrelation is the optimal method to minimize the linear effects of the PSA on measured eye position. However, it must be noted that even perfect decorrelation does not eliminate the PSA but only reduces it to a minimum. In addition, the regression method presented here was optimized for a specific frequency range, which was selected because the signal from the head-mounted pupil tracker used is only slightly distorted by head movements or tracker noise in this range. Of course, sources of error other than the PSA are not eliminated by the regression method. Head movements in the frequency range of interest may also contaminate the corrected measurement of fixation drift.

### Possible causes of the PSA

Different potential causes for the PSA have been discussed in the literature. Besides distortions in pupil shape (Wyatt, 1995), effects of optical refraction when observing the pupil through the cornea have been analyzed Hooge et al. (2021). According to this study, these optical effects (optical PSA) predict that, with pupil dilation, the pupil center appears deviated to more eccentric positions with a PSA gain that depends almost linearly on pupil position. Thus, the optical PSA is zero when the gaze is aligned with the optical axis of the camera and has the opposite sign for gaze directions to the right or to the left. The theoretical analysis (Hooge et al., 2021) of this gaze direction-dependent PSA gain showed that for horizontal gaze eccentricities smaller than 5 deg, the optical PSA gain is smaller than 0.015 deg/mm. The results of the current study differ from these theoretical predictions of optical PSA, as the PSA gains were more than ten times larger (0.23 deg/mm) and showed the same sign for gaze directions to the left and to the right (Fig. 3a). For gaze eccentricities below 5 deg, the direction-independent components of the PSA were much larger than the direction-dependent components. The predictions of the optical PSA in the vertical direction also do not correspond with the results of the current study. Because of the upward tilt of the camera’s optical axis in our setup, viewing directions on the horizontal meridian had an upward eccentricity of 30 degrees with respect to the camera. Optical PSA should therefore show upward pupil shift with pupil dilation and not the observed downward shifts. Thus, neither the horizontal nor the vertical observed PSA corresponded to the predictions of optical PSA. This result is in line with that of Hooge et al. (2021), who also found that the experimentally observed PSA cannot be explained by the optics of the cornea.

Genuine fixating eye movements, which would occur depending on pupil size, would be incorrectly interpreted as PSA by both correction methods examined here. Under binocular viewing conditions, coupling between pupil constriction and convergence could contribute to the PSA, as pupil constriction is associated with increased (convergent) fixation disparity (Jaschinski-Kruza, 1994). Under the monocular viewing conditions of our calibration paradigm, the coupling of pupil, accommodation, and vergence is also present. However, in monocular vision, changes in vergence only affect the non-seeing eye. Therefore, the PSA estimate of the seeing eye using the interpolation method in the calibration paradigm should remain unaffected by this coupling. In contrast, the regression method used under the binocular viewing conditions of the fixation paradigm could lead to an overestimation of the horizontal PSA gain. However, two observations argue against this. First, the two estimates did not differ significantly. Second, Fig. 4 of the study of Jaschinski-Kruza (1994) shows that a pupil dilation induced a divergent fixation disparity of only 0.3 arcmin/mm = 0.005 deg/mm, whereas the PSA in vergence is much larger with a gain of 0.5 deg/mm (Jaschinski, 2016). This value is consistent with twice the monocular PSA gain of 0.23 deg/mm, observed in the current study.

In summary, neither optical PSA nor true pupil fixational movements related to pupil size can explain the observed PSA. The third relevant factor for the PSA is the anisotropic dilation of the pupil that causes a physical shift of the pupil center with respect to the eyeball (Wyatt, 1995). Experiment 2 investigated the localization of the pupil center relative to the limbus center and its dependency on the pupil diameter. The results fully confirmed the results of Wyatt (1995) and refined them in that the current study used about 1500 images compared to 2 to 3 photographs per participant and background illumination. In addition, Experiment 2 showed that neither horizontal nor vertical PSA differed significantly between the camera and limbus coordinates. This supports the hypothesis that the physical shift of the pupil with respect to the eyeball induced by pupil dilation is the most relevant factor for explaining the PSA for small gaze eccentricities below 5 deg. The most important aspect of this observation is that it justifies the basic approach of the presented compensation methods to eliminate dependence of measured pupil position on pupil diameter because this dependence reflects movements of the pupil with respect to the eye rather than movements of the eye itself. This result is also relevant to eye-tracking technology because it supports the assessment of other studies (Strauch & Naber, 2022) that tracking the limbus and/or areas of the iris close to the limbus is less sensitive to PSA than tracking the edge of the pupil.

## Conclusion

Based on video recordings of a head-mounted pupil tracker, both the interpolation method and the regression method achieved similar reduction of the coefficient of determination between pupil diameter and eye position and were able to estimate the PSA gain with similar precision. However, with large coefficients of variation (horizontal: 39%; vertical: 15%), the precision of this estimate was rather poor. Advantages of the regression method include its invariance to changes in PSA across measurements and the need for no additional calibration records specific to different pupil diameters. The main advantage of the interpolation method is the capability to account for gaze direction-dependent components of the PSA gain. For investigating fixation drifts at a single fixation position at the center of the screen, as in the current experiment, this advantage seems of minor importance. Compensating fixation drifts for PSA seemed more important for vertical than for horizontal drift components since both PSA gains (horizontal: 0.23 deg/mm; vertical: 0.62 deg/mm) and the coefficients of determination between fixation drift and pupil diameter (horizontal: 1.5%; vertical: 14%) were larger for the vertical than for the horizontal direction. The successful reduction of these correlations demonstrated that measurements of fixation drift obtained with a head-mounted pupil tracker can be improved by compensating for the PSA.

With this compensation, spectral analysis of the fixation drifts evaluated in the frequency range of 1–10 Hz (Fig. 9) yielded results comparable to those obtained with the search coil or the dual-Purkinje-image techniques. This explicitly excludes the constant component of eye position. It is well known that this component can be measured much more accurately with the latter two methods than with head-mounted pupil trackers. Nevertheless, the presented PSA compensation is a first step toward making the field of fixation drift research in a narrow frequency range accessible to methods of pVOG.

## Supplementary Information

Below is the link to the electronic supplementary material.

Supplementary file1 (DOCX 152 kb)

## Data Availability

Data and code are available on the German Neuroinformatics Node platform: Eggert, T. (2026). Estimation of the pupil-size artifact to improve VOG measurements of fixation drift. G-Node. 10.12751/g-node.fr4nte.
