# Supplementary material for: Correction for the pupil size artifact improves the measurement of fixation drift with a head-mounted pupil tracker
Source: Behav Res Methods. 2026 Jul 14;58(8):234. doi: 10.3758/s13428-026-03103-z (PMC13369443; doi:10.3758/s13428-026-03103-z)
Supplement: Supplementary file 1 — Supplementary file1 (DOCX 152 kb) [file 13428_2026_3103_MOESM1_ESM.docx]

# Appendix

## Deriving vertical from horizontal linear calibration parameters

It has been shown previously (Ohlendorf et al., 2022) that, for a given meridian (vertical or horizontal), the rotation radius ($R$ [mm]) of the eye can be computed from the slope ($s$ [px/deg]) of a one-dimensional regression of pupil position [px] on target position [deg]:

|  | $R\cong\frac{s}{m} \frac{180}{\pi}$ | , | (Eq. A1) |
| --- | --- | --- | --- |

where $m$ [px/mm] denotes the magnification of the camera, defined as the ratio between the image size [px] and the object size [mm] (see Eq. 1). The rotation radius R is here defined as the distance between the center of rotation and the entrance pupil (the location of the virtual image of the pupil when seen through the cornea). Equation A1 follows from the fact that the inverse magnification 1/*m* is equal to the pupil shift [mm], which corresponds to one pixel in the camera. Such a pupil shift occurs for an eye position at the eccentricity $\alpha=1 [px]/s$. For small eccentricities $\alpha$, this pupil shift can be expressed using the rotation radius of the eye $R$ [mm]:

|  | $\frac{1}{m}=R sin\left( \frac{\pi}{180}\alpha\right)=R sin\left( \frac{\pi}{180}\frac{1}{s} \right)\cong\frac{R}{s}\frac{\pi}{180}$ | . | (Eq. A2) |
| --- | --- | --- | --- |

Solving Eq. A2 for $R$ leads to Eq. A1. For cameras with isotropic optics, the magnification factor $m$ is the same for the vertical and horizontal meridians. Therefore, the ratio between the vertical and horizontal regression slope is identical to the ratio between the vertical and horizontal rotation radius of the eye:

|  | $\frac{s_{V}}{s_{H}}=\frac{R_{V}}{R_{H}}$ | . | (Eq. A3) |
| --- | --- | --- | --- |

Because the ratio between the vertical and horizontal rotation radius is about 0.76 (Ohlendorf et al., 2022), Eq. A3 was used to compute an estimate of the vertical regression slope ($\hat{s}_{V}$) from the horizontal one:

|  | $\hat{s}_{V}=0.766 s_{H}$ | . | (Eq. A4) |
| --- | --- | --- | --- |

To test whether this procedure (Eq. A4) allows reliable estimation of the vertical from the horizontal regression slope, we analyzed calibration data from another (unpublished) experiment in which 11 participants performed a full 2D-calibration paradigm with a 3 × 3 target grid (± 15 deg) and with seven fixations per target. The two regression slopes $s_{H}$ and $s_{V}$ and the corresponding horizontal and vertical rotation radii $R_{H}$=10.11 ± 0.91 mm and $R_{V}$=7.74 ± 0.91 mm were calculated from these data according to Eq. A1. Ohlendorf et al. (2022) reported slightly larger rotation radii ($R_{H}$= 11.7 ± 1.5 mm; $R_{V}$ = 8.9 ± 1.4 mm). However, their ratio (0.761) was practically identical to that observed in the current study (0.766), shown in Fig. A1. Moreover, the linear regression of $R_{V}$ on $R_{H}$ (Fig. A1 solid) was very close to the line connecting the origin of the coordinate system and the mean (Fig. A1, dashed). The Pearson correlation between $R_{V}$ and $R_{H}$ (not reported by previous studies) was highly significant ($\rho$ = 0.82; *p* = 0.002). Taken together, these results suggest that the main differences in rotation radius between subjects are explained by a subject-specific scaling factor that applies to both the horizontal and vertical dimensions of the eye.


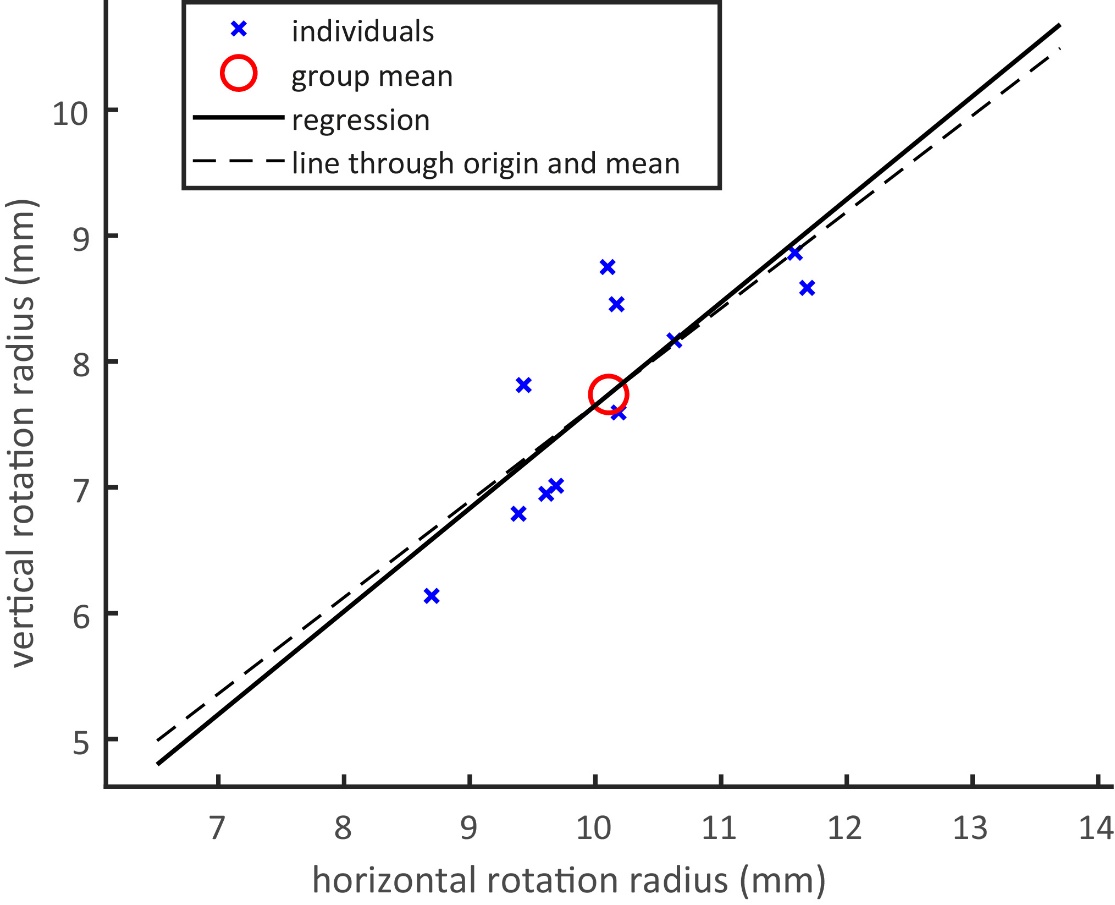

**Figure****A1** Horizontal and vertical rotation radii ($R_{H}$, $R_{V}$) of 11 subjects (*blue crosses*). *Red circle*: group mean. *Black solid*: The regression of the vertical on the horizontal rotation radius ($\hat{R}_{V}=$– 0.533 + 0.82 $R_{H}$). Its offset (– 0.533) did not differ significantly (T(9) = – 0.28; *p* = 0.79) from zero, and its slope (0.82) did not differ significantly from the ratio between the vertical and horizontal mean (${\bar{R}_{V}}/{\bar{R}_{H}=0.766};T\left( 9 \right)=0.28;p=0.77).$ Therefore, estimation of the vertical rotation radius by $\hat{R}_{V}=0.766 R_{H}$ (*dashed line*) is unbiased. Its percentage error ($100(1-{\hat{R}_{V}}/{R_{V}}$)) had a standard deviation of 7%

Figure A1 shows that the estimation of the vertical rotation radius $\hat{R}_{V}=0.766 R_{H}$ has two important properties: First, it is unbiased, and second, it has a standard deviation of only 7% of its mean, i.e., a coefficient of variation of 0.07. The same two properties also apply to the estimate $\hat{s}_{V}$ of the vertical regression slope (Eq. A4) because $R_{V}$ and $s_{V}$ are proportional to each other (Eq. A1). This means that the error that results from estimating the regression slope $s_{V}$ using $s_{H}$ instead of performing a full vertical calibration is mean-free and has a standard deviation of only 7% of the true slope.

## **Extracting slow-phase components of eye position**

The process to exclude microsaccades and frequency components above 15 Hz from the eye position (Eq. 12) generates the *slow-phase of eye position* ($spe\left( t \right)$). This signal was extracted for multiple purposes. First, for estimating the PSA using the regression method, and second, for evaluating the fixation drift after PSA correction was performed. The calculation was performed in the following steps: a) computing eye velocity; b) selecting fixation intervals; c) detection of micro-saccades; d) low-pass filtering of eye position in the intersaccadic intervals with a spline filter (3-dB cutoff frequency: 10 Hz, 40-dB cutoff frequency: 15 Hz); e) calculating the slow-phase components of eye velocity by differentiation of the spline-filtered intersaccadic eye position and linear interpolation between start and end of saccades; f) integration of slow-phase eye velocity to obtain the slow-phase position. These six steps are explained in detail below.

a) Horizontal and vertical components of eye velocity were calculated with a three-point differentiator applied after a Gaussian low-pass filter with a cutoff frequency of 33 Hz (transmission gain of 0.1 at 85 Hz) and zero phase delay.

b) All fixation intervals had a duration between 1 s and 2 s and were selected according to the following criteria: The interval did not contain missing values, the maximum eye displacement within the interval stayed below 1.6 deg, and eye velocities stayed below 15 deg/s for at least 90% of the interval. From the fixation paradigm, 76 ± 22 fixation intervals were selected from the periods between the button press and the onset of the Landolt-C in the following fixation trial. From the calibration paradigm, 75 ± 26 fixation intervals were selected from the periods between the target steps.

c) Since these selected fixation intervals could still contain microsaccades and artifacts due to partial blinks, they were fully segmented, with each subsegment belonging to either a microsaccade or an artifact (non-fixation segment) or not (fixation segment). The non-fixation segments were detected with an algorithm developed and used in previous studies (Ladda, Eggert, Glasauer, & Straube, 2007; Nuding, Ono, Mustari, Buttner, & Glasauer, 2008). This algorithm assumes that microsaccades and blink artifacts cause fast velocity components that are additively superimposed on the slow-phase component. A non-fixation segment was detected whenever the fast velocity component increased above 15 deg/s. The start and end of these non-fixation segments were defined by the times before and after peak velocity when the fast-phase velocity vector either became zero or deviated more than 90 deg from its direction at peak velocity.

d) The eye position within all fixation segments was smoothed with a cubic spline filter. The support values of the spline minimized the squared difference between the eye position and the spline interpolation. This minimization was performed under the boundary constraints that the initial and final spline velocity equaled the mean eye velocity during the first and last interpolation interval (i.e., the interval between the support points of the spline). The interpolation interval was 47 ms. This (time-variant, linear) filter is a zero-phase filter, characterized by a cutoff frequency of 10 Hz and a gain of 0.01 at 15 Hz. Thus, it effectively excludes frequencies above 15 Hz from the analysis of fixation drift. This method allows selective cleaning of intersaccadic intervals using a relatively strong low-pass filter without distorting the timing and dynamics of saccades. Conversely, the filtered intersaccadic intervals remain unaffected by the saccades.

e) The slow-phase components of eye velocity were obtained by differentiation of the spline-filtered eye position during the fixation segments and linear interpolation across the non-fixation segments.

f) Finally, the slow-phase position ($spe\left( t \right)$) of each fixation interval was calculated by integration of the slow-phase velocity, numerically approximated by the Riemann integral, and re-centered to its mean (subtractive normalization). We define 'fixation drift' as the slow-phase eye position calculated after PSA correction.

## **Effectiveness of PSA correction**

The coefficient of determination ($R^{2}$) of the linear regression of slow-phase eye position on pupil diameter was evaluated for each participant and for each fixation interval of the fixation paradigm. It expresses the fraction of the variance of slow-phase eye position explained by changes in pupil size. As with the calculation of the regression (Eq. 13), the coefficient of determination was also calculated in the spectral domain. Frequencies below 1 Hz were excluded (see section *Spectral analysis*). For each participant, the mean coefficient of determination ($\overline{R}^{2}$), averaged across all fixation intervals, is an estimate of the fraction of apparent fixation drift predictable by pupil diameter. It was calculated in three different ways. First, after calibration, while ignoring the pupil diameter (Eq. 12). This is a measure of the relative contribution of the PSA to the uncorrected drift measurement. Second, after correcting for the PSA with the interpolation method, and third, after correcting with the regression method. The last two $\overline{R}^{2}$ values estimate the remaining relative contribution of the PSA after applying one or the other method. Their difference is used to compare the effectiveness of the two methods.^[[1]](#footnote-1)^

## **Spectral analysis**

To calculate the regression slope $\beta_{k}$ (Eq. 13) in each fixation interval *k*, the slow-phase eye position ${spe}_{k}(n \Delta t)$ and the pupil diameter ${pd}_{k}\left( n \Delta t \right)$ were submitted before PSA correction to a spectral analysis. For the sake of brevity, we define for this section

|  | $x_{k}\left( n \Delta t \right)={pd}_{k}\left( n \Delta t \right), y_{k}\left( n \Delta t \right)={spe}_{k}(n \Delta t)$ | , | (Eq. A5) |
| --- | --- | --- | --- |

and the periodograms $S_{xx}^{k}, S_{yy}^{k}$, and $S_{xy}^{k}$ using the Hann window (Blackman & Tukey, 1958, pp. 200–201) with the width of the shortest fixation interval ($T_{H}$=1 s) and zero padding to the duration of the longest fixation interval ($T$= 2 s):

|  | | $S_{xx}^{k}\left( m \Delta f \right)=\frac{\Delta t}{Nf}\left\vert\sum_{n=0}^{N-1} hann\left( \frac{n \Delta t}{T_{H}} \right)x_{k}\left( n \Delta t \right) exp\left( -i2\pi\frac{n m}{N} \right) \right\vert^{2}$ | | |  | | (Eq. A6a) |
| --- | --- | --- | --- | --- | --- | --- | --- |
|  | | $S_{yy}^{k}\left( m \Delta f \right)=\frac{\Delta t}{Nf}\left\vert\sum_{n=0}^{N-1} hann\left( \frac{n \Delta t}{T_{H}} \right)y_{k}\left( n \Delta t \right) exp\left( -i2\pi\frac{n m}{N} \right) \right\vert^{2}$ | | | , | | (Eq. A6b) |
|  | | $S_{xy}^{k}\left( m \Delta f \right)=\frac{\Delta t}{Nf}\left[ \sum_{n=0}^{N-1} hann\left( \frac{n \Delta t}{T_{H}} \right)x_{k}\left( n \Delta t \right) exp\left( -i2\pi\frac{n m}{N} \right) \right]^{*}$ | | |  | | (Eq. A6c) |
|  | | | $\cdot\left[ \sum_{n=0}^{N-1} hann\left( \frac{n \Delta t}{T_{H}} \right)y_{k}\left( n \Delta t \right) exp\left( -i2\pi\frac{n m}{N} \right) \right]$ | | , | |  |
|  | $\text{with} N=floor\left( \frac{T}{\Delta t} \right)\text{ }\text{,} hann\left( x \right)=\left\{ \begin{matrix} {sin}^{2}\left( \pi x \right) & \text{for} 0<x<1 \\ 0 & \text{for} x> 1 \end{matrix}\begin{matrix} \\ \end{matrix} \right.$ | | | | , | | (Eq. A6d) |
|  | $\text{and the normalization factor}\text{ }\text{ }Nf=\sum_{n=0}^{N-1} {hann}^{2}\left( \frac{n \Delta t}{T_{H}} \right)$ | | |  | | . | (Eq. A6e) |

The spectral sampling interval is $\Delta f=1/T$, and $\left[ \ldots\right]^{*}$ denotes the complex conjugate. Using these definitions, the covariance between filtered eye position and filtered pupil diameter in the fixation interval k was calculated by

|  | $cov\left( \underline{fe}_{k}, \underline{fpd}_{k} \right)=\frac{1}{\left( N-1 \right) \Delta t} \sum_{m=2}^{N-2} S_{xy}^{k}(m \Delta f)$ | , | (Eq. A7) |
| --- | --- | --- | --- |

and the variance of the pupil diameter by

|  | $var\left( \underline{fpd}_{k} \right)=\frac{1}{\left( N-1 \right) \Delta t} \sum_{m=2}^{N-2} S_{xx}^{k}(m \Delta f)$ | . | (Eq. A8) |
| --- | --- | --- | --- |

The regression slope $\beta_{k}$ was then obtained by inserting Eqs. A7/A8 into Eq. 13. The fact that the index range ($2\leq m\leq N-2$ in Eqs. A7/A8) excluded the fundamental frequency ($f=1/T$) ensured that the estimation of the regression slope was only minimally affected by frequency components below 1 Hz ($=2/T$).

Spectral analysis was also used to calculate the coefficient of determination ($R^{2}$) of the linear regression of slow-phase eye position on pupil diameter:

|  | $R^{2}=\frac{{cov\left( \underline{fe}_{k}, \underline{fpd}_{k} \right)}^{2}}{var\left( \underline{fe}_{k} \right) var\left( \underline{fpd}_{k} \right)}$ | . | (Eq. A9) |
| --- | --- | --- | --- |

Frequencies below 1 Hz were excluded by inserting Eqs. A7, A8 and

|  | $var\left( \underline{fe}_{k} \right)=\frac{1}{\left( N-1 \right) \Delta t} \sum_{m=2}^{N-2} S_{yy}^{k}(m \Delta f)$ |  | (Eq. A10) |
| --- | --- | --- | --- |

into Eq. A9. In this way, the signal components used to evaluate the coupling between pupil position and pupil diameter were the same as those used to estimate PSA in the regression method.

After the PSA correction by the regression method, spectral analysis was also applied to the slow-phase eye position ${spe}_{k}(n \Delta t)$ to decompose the alternating power of the fixation drift in its frequency components. The power spectral density of the measured fixation drift (${PSD}_{drift}$) was estimated according to Bartlett’s method (Bartlett, 1948) by averaging the periodograms across all fixation intervals of a participant (*K* = 76 ± 22):

|  | ${PSD}_{drift}\left( k \Delta f \right)=\frac{1}{K}\sum_{k=1}^{K} S_{yy}^{k}\left( m \Delta f \right)$ | . | (Eq. A11) |
| --- | --- | --- | --- |

Due to the symmetry properties of ${PSD}_{drift}\left( m \Delta f \right)$, the variance of the fixation drift is

|  | $var\left( spe \right)=\frac{1}{\left( N-1 \right) \Delta t} \sum_{m=1}^{N-1} {PSD}_{drift}(m \Delta f)$ | |  | (Eq. A12) |
| --- | --- | --- | --- | --- |
|  | | $=\frac{1}{\left( N-1 \right) \Delta t} \sum_{m=1}^{floor\left( \frac{N}{2} \right)} 2 {PSD}_{drift}(m \Delta f)$ | , |  |

and is therefore commonly plotted only for $m\leq floor\left( \frac{N}{2} \right)$, i.e., for the positive frequencies. In accordance with prevailing convention, Fig. 9 thus illustrates $2 {PSD}_{drift}(m \Delta f)$. Consequently, in a magnitude plot with linear-linear coordinates, the area beneath this curve is equivalent to the variance of the fixation drift.

## **References**

Bartlett, M. S. (1948). Smoothing periodograms from time-series with continuous spectra. *Nature, 161*(4096), 686–687. doi:10.1038/161686a0

Blackman, R. B., & Tukey, J. W. (1958). The measurement of power spectra from the point of view of communications engineering—Part I. *Bell System Technical Journal, 37*(1), 185–282.

Ladda, J., Eggert, T., Glasauer, S., & Straube, A. (2007). Velocity scaling of cue-induced smooth pursuit acceleration obeys constraints of natural motion. *Experimental Brain Research, 182*(3), 343–356. Retrieved from PM:17562031

Nuding, U., Ono, S., Mustari, M. J., Buttner, U., & Glasauer, S. (2008). A theory of the dual pathways for smooth pursuit based on dynamic gain control. *J Neurophysiol, 99*(6), 2798–2808. doi:10.1152/jn.90237.2008

Ohlendorf, A., Schaeffel, F., & Wahl, S. (2022). Positions of the horizontal and vertical centre of rotation in eyes with different refractive errors. *Ophthalmic and Physiological Optics, 42*(2), 376–383. doi:10.1111/opo.12940

1. Note that this difference in $\overline{R}^{2}$ only evaluates the compensation of the linear dependencies of the apparent fixation drift on the pupil diameter. In this respect, it corresponds to the regression method, which in the form used here only takes such linear dependencies into account. The underlying assumption of linearity appears to be approximately fulfilled for changes in pupil diameter of less than 2 mm, but may also be violated in individual cases (see Hooge et al., 2021, Fig. 3). [↑](#footnote-ref-1)
